# MiR-200c-3p maintains stemness and proliferative potential in adipose-derived stem cells by counteracting senescence mechanisms

**DOI:** 10.1371/journal.pone.0257070

**Published:** 2021-09-17

**Authors:** Eleni Anastasiadou, Simona Ceccarelli, Elena Messina, Giulia Gerini, Francesca Megiorni, Paola Pontecorvi, Simona Camero, Maria Giuseppina Onesti, Pankaj Trivedi, Mario Faenza, Enrico Coscioni, Giovanni Francesco Nicoletti, Claudio Napoli, Cinzia Marchese

**Affiliations:** 1 Department of Experimental Medicine, Sapienza University of Rome, Rome, Italy; 2 Department of Maternal, Infantile and Urological Sciences, “Sapienza” University of Rome, Rome, Italy; 3 Plastic Surgery Unit, Department of Surgery "P. Valdoni", Sapienza University of Rome, Rome, Italy; 4 Multidisciplinary Department of Medical, Surgical and Dental Sciences, Plastic Surgery Unit, University of Campania "Luigi Vanvitelli", Naples, Italy; 5 Department of Cardiac Surgery, Azienda Ospedaliera Universitaria San Giovanni di Dio e Ruggi d’Aragona, Salerno, Italy; 6 Department of Advanced Medical and Surgical Sciences (DAMSS), University of Campania "Luigi Vanvitelli", Naples, Italy; Government College University Faisalabad, PAKISTAN

## Abstract

Adipose-derived mesenchymal stem cells (ASCs) are promising therapeutic tools in regenerative medicine because they possess self-renewal, differentiation and immunomodulatory capacities. After isolation, ASCs are passaged multiple times *in vitro* passages to obtain a sufficient amount of cells for clinical applications. During this time-consuming procedure, ASCs become senescent and less proliferative, compromising their clinical efficacy. Here, we sought to investigate how *in vitro* passages impact ASC proliferation/senescence and expression of immune regulatory proteins. MicroRNAs are pivotal regulators of ASC physiology. Particularly, miR-200c is known to maintain pluripotency and targets the immune checkpoint Programmed death-ligand 1 (PD-L1). We therefore investigated its involvement in these critical characteristics of ASCs during *in vitro* passages. We found that when transiently expressed, miR-200c-3p promotes proliferation, maintains stemness, and contrasts senescence in late passaged ASCs. Additionally, this miRNA modulates PD-L1 and Indoleamine 2,3-Dioxygenase (IDO1) expression, thus most likely interfering with the immunoregulatory capacity of ASCs. Based on our results, we suggest that expression of miR-200c-3p may prime ASC towards a self-renewing phenotype by improving their *in vitro* expansion. Contrarily, its inhibition is associated with senescence, reduced proliferation and induction of immune regulators. Our data underline the potential use of miR-200c-3p as a switch for ASCs reprogramming and their clinical application.

## Introduction

Adipose-derived mesenchymal stem cells (ASCs) represent a population of self-renewing multipotent adult cells. They are isolated from the vascular stroma of adipose tissue.

The main biological properties of ASCs useful in cell-based therapy are: 1) the ability to proliferate and to maintain their stemness. These are important characteristics for the maintenance of a relatively stable clone of self-renewing cells within the target tissue, 2) the expression of immunomodulatory proteins on the surface of ASCs or secreted enzymes, in the so-called secretome, which enable them to escape immune recognition. ASCs are able to improve the microenvironment for tissue healing through strong immunosuppressive functions by decreasing inflammatory cytokine production. Thus, ASCs can be used in the clinical treatment of inflammatory and autoimmune pathologies, such as multiple sclerosis, inflammatory bowel disease, graft-versus-host disease (GvHD), rheumatoid arthritis (RA) and other diseases [1–5].

One of the main caveats for ASCs clinical applications is that they often lose the stem cell niche environmental protection and sustainment after their isolation and *in vitro* expansion. Stemness is important to maintain ASCs pluripotency, but also their immunomodulatory properties.

Freshly isolated human ASCs express the pluripotency markers Nanog, Oct4 and Sox2 during *in vitro* culture but their expression gradually decreases. This leads to stemness loss and senescence of ASCs, with limitations of proliferation/population doubling, this being a major hindrance in ASC-based therapy [6]. The replicative senescence is regulated by the p53/p21 pathway, which can be differentially expressed during the *in vitro* passaging along with the stemness markers [7]. Moreover, there is a plethora of epigenetic-sensitive paths involved in ASC differentiation [8]. Even the immunosuppressive ability of ASCs is cell passage dependent [9]. Critically, during serial passaging, ASCs start losing their immunosuppressive properties. For instance, expression of Major Histocompatibility Complex, Class I, G (HLA-G), a non-classical MHC-class I molecule, decreases in adult ASCs together with immunotolerance through inhibition of NK, allogeneic T-cell responses and dendritic cells (DC) [10]. HLA-G is also involved in epigenetic regulation of tolerance to cardiac ischemia as well as susceptibility to infection after kidney transplantation [11–13]. A well-known immune checkpoint (IC) with immunosuppressive functions is the Programmed death-ligand 1 (PD-L1) [14]. It binds to its ligand PD-1 on T-cells and suppresses T cell immune response [15]. PD-L1 function has been extensively studied on human mesenchymal stem cells from placenta, umbilical cord blood and bone marrow, but little is known about its expression and function in ASCs [16]. ASCs also secrete the catabolic enzyme IDO1, which inhibits immune response and tissue inflammation [17]. IDO1 catalyzes the cleavage of the essential amino acid tryptophan into “Kynurenines” which leads to important immunomodulation [17]. Over-expression of IDO1 induces graft tolerance and attenuates acute rejection of tissue-engineered lung allografts in rats [18]. Thus, expression of co-inhibitory molecules, such as PD-L1, as well as anti-inflammatory enzymes like IDO1, might contribute to immunomodulation of ASCs during in *vitro* expansion [16, 19–21]. To date, the role of the ICs in ASC-mediated immunomodulation has not been thoroughly investigated.

MicroRNAs (miRNAs) 18–25 nucleotides long, endogenous, noncoding RNAs that post-transcriptionally regulate gene expression through complementary binding of the so called seed sequence of miRNA to the microRNA response element (MRE) on 3’UTR of the target mRNA [22]. It is now established that miRNAs play a critical role in the maintenance, differentiation, and lineage commitment of stem cells in various cellular processes [23–25]. MiR-200c, one of the five members belonging to miR-200 family, regulates the mesenchymal-to-epithelial transition (MET) [26]. There is an inverse correlation between miR-200c and Nanog in cancer [27]. Contrarily, in induced pluripotent stem cells (iPSC), Oct4 and Sox2 directly binds and activates the promoters regions of the miR-200 family members, promoting iPSC regeneration [23]. In addition, the knockdown of miR-200c downregulates Nanog/c-Myc expression and inhibits embryonic stem cells renewal, thus indicating to the ability of miR-200c to maintain stem cells pluripotency and self-renewal properties [28].

Here, we investigated the role of miR-200c-3p in ASCs stemness, proliferation and senescence during *in vitro* expansion. Furthermore, to better understand the role of PD-L1 in immunomodulation, its expression was analyzed in serially passaged ASCs.

## Materials and methods

### Cell isolation and culture

ASCs were isolated from abdominal subcutaneous fat liposuction aspirates of five healthy donors who underwent elective plastic surgery. Liposuction aspirates were transferred to the laboratory and processed under sterile conditions within 24 h. Isolation and phenotypic characterization of ASCs were performed as previously described [8]. Briefly, liposuction aspirates were washed extensively with sterile phosphate-buffered saline (PBS) containing 2% penicillin/streptomycin (PS) and subjected to enzymatic digestion with 0.075% collagenase type I for 30–60 min at 37°C and 5% CO_2_. The suspension was filtered through a 100-μm mesh filter to remove debris and centrifuged for 5 min at 2000 rpm. The pellets of stromal vascular fraction (SVF) containing ASCs were washed with PBS, resuspended in DMEM-Ham’s F-12 (v/v, 1:1) (DMEM/F12; Gibco) supplemented with 10% FBS, 100 U/ml penicillin, 100 μg/ml streptomycin, and 2 mM l-glutamine and plated in a 75-cm^2^ tissue culture flask coated with collagen (type IV; Sigma-Aldrich, Milan, Italy). ASCs were self-selected out of the SVF, since they were adherent to the plastic tissue cultureware. ASCs were maintained in a 5% CO_2_ incubator at 37°C in a humidified atmosphere, with medium change twice a week. At 80–90% confluence, cells were detached with 0.5 mM EDTA/0.05% trypsin (Gibco) for 5 min at 37°C and replated. ASCs were expanded and the cell viability was assessed using the trypan blue exclusion assay. Cell morphology was evaluated by phase contrast microscopy. A homogeneous population of ASCs was subsequently checked by analyzing the surface marker expression profile, as previously described [29]. Experiments were conducted at passage numbers from 2 to 15, as indicated. The absence of mycoplasma contamination was confirmed by PCR with specific primers.

### Statement of research involving human subjects

The use of clinical samples of adipose tissue for ASCs isolation were complied with the Declaration of Helsinki 1975, revised in 2008, and the study methodologies have been approved by the Institutional Review Board of the Department of Experimental Medicine of the Sapienza University of Rome. The experiments were undertaken with the understanding and written fully informed consent of each subject.

### MiR-200c-3p mimic and inhibitor transfections

One day before transfection, ASCs at P4 or P9 were seeded in 6 well-plate at a density of 0.3 × 10^5^ cells/well. Transfection was performed in triplicates with 40 nM of mimic miR-200c-3p or mimic control for cells at P4 and with miR-200c-3p inhibitor or inhibitor control for cells at P9 (MISSION®, Sigma-Aldrich). The compounds were delivered into the cells using DharmaFect Duo transfection reagent (Dharmacon, Horizon a PerkinElmer company, Diatech, Jesi, Italy) following the manufacturer’s instructions. After 72 h, cells were harvested for total RNA and protein extraction.

### Protein extracts and Western blot

Total cell extracts from 0.8–1.0x10^5^ ASCs and miR-200c-3p transfectants were prepared by RIPA lysis. 15 μg of proteins were resolved on SDS-PAGE and transferred to PVDF membranes.

The membranes were blocked with 5% non-fat dried milk for 1h and incubated overnight at 4°C and 1 h at room temperature with the appropriate primary antibody. Subsequently, HRP-conjugated anti-rabbit (ADVANSTA, San Jose, CA, USA) [1:10000 dilution] and anti-mouse (BETHYL) [1:40000 dilution] secondary antibodies were used. The chemiluminescent signal was detected using WesternBright ECL HRP substrate kit (ADVANSTA). Blots were probed with the following antibodies: PD-L1 E1L3N (Cell signaling Technology, Danvers, MA, USA) [1:1000 dilution], cyclin D1 (DCS-6) (Santa Cruz Biotechnology, Inc., Heidelberg, Germany) [1:500 dilution], p53 (DO-1) (Santa Cruz) [1:300 dilution], p21 (F-5) (Santa Cruz) [1:100 dilution], p-ERK (E-4) (Santa Cruz) [1:600 dilution], ERK 2 (C-14) (Santa Cruz) [1:300 dilution], CD44 (156-3C11) (Invitrogen, Thermo Fisher Scientific) [1:2500 dilution] P-STAT3 (Tyr705) (D3A7) XP (Cell signaling) and STAT3 (clone 84) (BD Transduction Lab.). β-actin (C4) (Santa Cruz) [1:5000 dilution] was used to ensure equal protein loading. Immunoblottings were repeated at least three times for each experimental conditions.

Densitometry analysis was performed with ImageJ Software (v.10.2). The number of pixels from each protein signal imprinted on a film was normalized to the number of pixels of the housekeeping β-actin, calculated as a ratio.

### RNA extraction

Total RNA from ASCs was extracted using 0.5 ml of TRIzol™ reagent (Invitrogen, Milan, Italy), according to the manufacturer’s instructions. RNA samples obtained with phenol-chloroform extraction were quantified using a MaestroNano micro-scale spectrophotometer (MaestroGen Inc.) and stored at -80°C, as described [30].

### Quantitative Real Time PCR (qRT-PCR)

A quantity of 0.5 μg RNA extracted from ASCs were reverse transcribed in a BioRad MyCycler Thermal Cycler machine, with miScript II RT Kit (QIAGEN S.r.l., Milan, Italy) according to the manufacturer’s instructions, for the subsequent detection of miRNAs and mRNAs from the same cDNA. Detection of PD-L1 and GAPDH mRNAs was performed with QIAGEN QuantiTect Primer Assay predesigned primers (Hs_CD274_1_SG QuantiTect Primer Assay, Hs_GAPDH_1_SG QuantiTect Primer Assay). Amplification of p53, p21, IDO1 and GAPDH mRNA was done using predesigned KiCqStart SYBR® Green primers (Sigma-Aldrich, Inc.), while the primers sequences for Nanog, Oct4, Sox2 and CD44 amplification were previously published [31].

Mature miR-200c-3p and U6 expression levels were detected using miScript Primer Assay (QIAGEN; Hs_miR-200c-3p_1 miScript Primer Assay, Hs_RNU6-2_11). Quantitative PCRs were performed using SYBR®Green PCR Kit (QIAGEN) in an Applied Biosystems/StepOne Software v2.2.2 QPCR machine. The fold change was calculated by the 2^-ΔΔCt^ formula and the experiments were repeated at least three times and in three technical replicates.

### Colony formation assay

ASCs transfected either with miR-200c-3p mimic or inhibitor and their respective controls were seeded in triplicates, at 72 h post-transfection, in 6-well plates at a density of 2 × 10^3^ cells/well. The plates were incubated at 37°C for 14–20 days, in order to allow colonies growth. Growth medium was changed every 3 days. Colony formation assays were performed as previously described [31, 32] and repeated at least three times. Briefly, colonies were stained with 0.1% crystal violet for 10 min at room temperature and photographed. Then, crystal violet was solubilized in 30% acetic acid in water for 15 min at RT, and absorbance was measured using the Biochrom Libra S22 UV/VIS spectrophotometer (Biochrom, Berlin, DE) at a wavelength of 595 nm. 30% acetic acid in water was used as blank control. Colony formation capacity in mimic/anti-miR transfected cells was calculated in comparison to control mimic/inhibitor transfected samples, arbitrarily set to 1.

### Senescence-associated β-Galactosidase assay

Senescence associated (SA)-β-Galactosidase staining was performed using the Senescence β-Galactosidase Staining Kit (Cell Signaling Technology) following the manufacturer’s instructions. Briefly, 72 h post-transfections, ASCs were washed with PBS and fixed in fixative solution for 15 minutes at room temperature. Cells were stained with the SA-β-Galactosidase staining solution for 24 h, at 37°C. Increased endogenous β-galactosidase activity at pH 6.0, used to determine the state of senescence, was shown by the development of a blue colour. The percentage of SA-β-galactosidase-positive cells was determined by counting blue-stained cells and the total number of cells per field in at least 7–8 different areas and in triplicates for each condition. Before staining, images of living cells were taken at 72 h post-transfection with EVOS XL Core Imaging System (ThermoFisher Scientific) at 40x magnification.

### Statistical analysis

Statistical analyses were done using Prism 7 software. Statistical significance (p values) of the results obtained by qRT-PCR and densitometry analyses of WBs were calculated using two-tailed unpaired t tests, ordinary one-way Anova or Dunnett’s multiple comparisons test. Statistical significance for colony formation and SA-β-Galactosidase staining was performed by two-tailed unpaired t tests. Correlation analysis was performed using Pearson correlation coefficients, such as, square of correlation coefficient, R^2^, p value was calculated as two-tailed and the confidence internal was set at 95%. The level of statistical significance was set at p < 0.05, for all experiments.

## Results

### MiR-200c-3p and stemness markers during *in vitro* passages of ASC

We sought to investigate the expression of miR-200c-3p in ASCs and its possible modulation during *in vitro* passages. For the experiments we have selected ASCs from passage P2. These cells expressed high levels of CD29, CD90, CD166, CD44 mesenchymal stemness markers, but did not express hematopoietic markers, CD34 and CD45 (S1 Fig).

**Fig 1:**
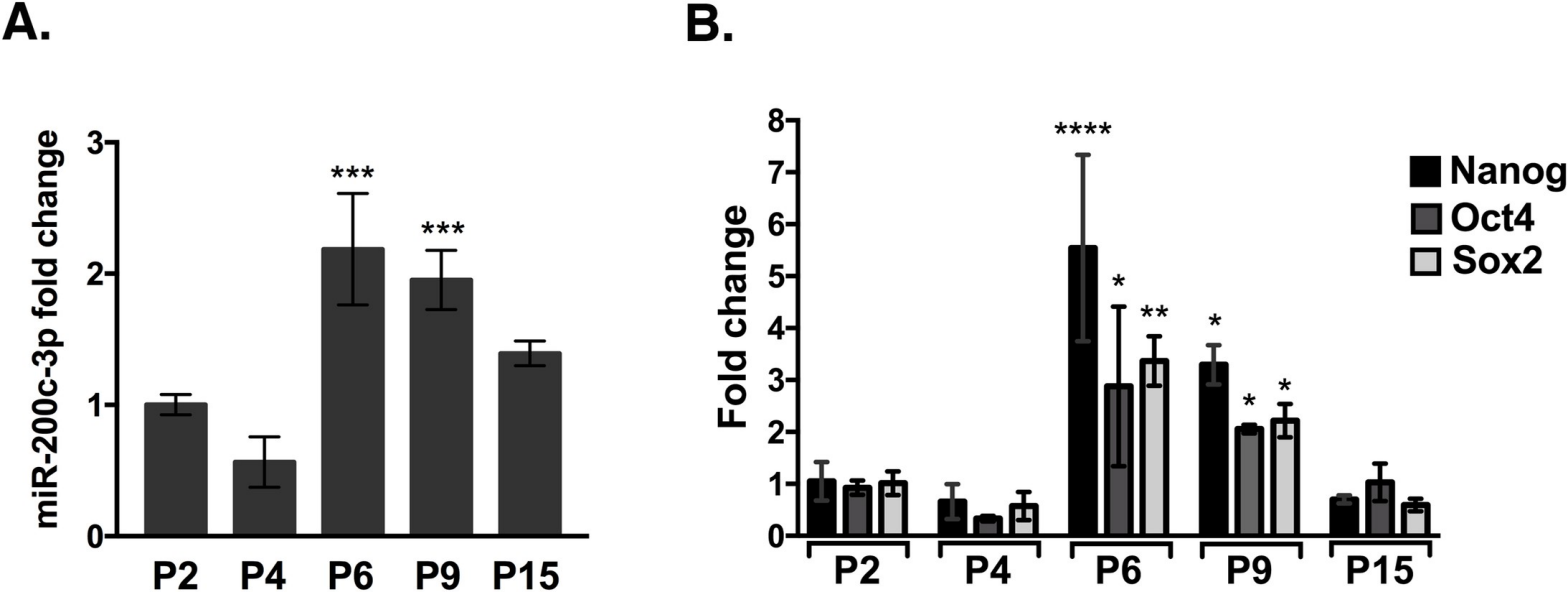
MiR-200c-3p and stemness markers expression during *in vitro* passages in ASCs. **A.** MiR-200c-3p expression by qRT-PCR, during *in vitro* passages (P). MiR-200c-3p expression was normalized to U6. SD± are the mean of three independent experiments. Dunnett’s multiple comparisons test was applied to compare P2 with each of the passages. *p<0.05, **p<0.01, ***p<0.001. **B.** Stemness markers during the *in vitro* passages were normalized to GAPDH. SD± are the mean of three independent experiments. Ordinary one-way Anova, Dunnett’s multiple comparisons test was performed for each stemness marker and p values were calculated comparing P2 with the rest of the passages. *p<0.05, **p<0.01, ****p<0.0001.

Fig 1A shows the expression of miR-200c-3p in ASCs at passages 2, 4, 6, 9 and 15. The data suggest a variable expression trend, with a reduction at P4, an increase at P6, P9 and a return to basal levels at P15 (Fig 1A). The difference of miR-200c-3p expression between P6 and P9 was not statistically significant, thus for further experiments we used ASCs at P9. Next, we assessed the expression of stemness markers Nanog, Oct4 and Sox2 in relationship with miR-200c-3p expression. A concomitant increase of Nanog, Oct4 and Sox2 at P6 and P9 was followed by a return to basal levels at P15 (Fig 1B). These results suggest to indicate a positive correlation between miR-200c-3p expression and stemness in ASCs.

### Proliferation and senescence markers are modulated in ASCs during *in vitro* culture

**Fig 2:**
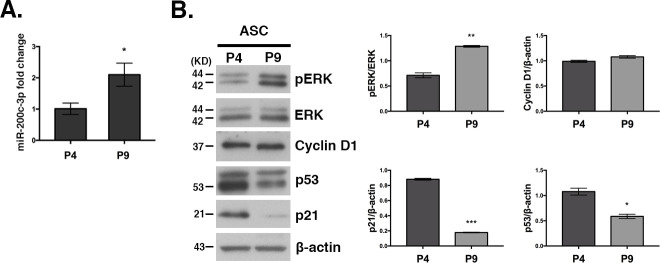
Expression of proliferation/senescence markers at P4 and P9 passages in ASCs. **A.** Expression analysis of miR-200c-3p at P4 versus P9 was performed by qRT-PCR and calculated as a fold change using 2^-ΔΔCt^. SD± is the mean of three experiments and the p values were calculated with two-tailed unpaired t test, *p<0.05, ***p<0.001. **B.** Western blot analysis of the proliferation-related markers p-ERK, ERK, cyclin D1, and the senescence-related markers p53 and p21, at P4 and P9. β-Actin was used as loading control. WBs were repeated twice. Densitometric analysis was performed with ImageJ Software (v.10.2) for each protein. Normalization was calculated as a ratio between the number of pixels (n.p.) of p-ERK divided to the n.p. of ERK and the rest of the proteins normalized with β-actin. SD± is the mean of two WBs and the p values were calculated with two-tailed unpaired t test, *p<0.05, **p<0.01, ***p<0.001.

The observation of increased stemness markers expression at P4 vs P9 (Fig 1B) seems inconsistent with the assumption of a physiological decrease of ASCs proliferation and stemness and a parallel increase of senescence during *in vitro* passages [33]. So, we sought to investigate these two characteristics in ASCs, at P4 and P9 passages where the switch in miR-200c-3p expression occurs (Fig 2A). We assessed the expression of phosphorylated Extracellular Signal-Regulated Kinase (p-ERK) and Cyclin D1, which are important regulators of ASC proliferation [34]. Since p53/p21 axis antagonizes self-renewal and pluripotency in human embryonic stem cells and high expression of these markers is associated with cellular senescence [35], their expression was also analyzed in ASCs.

The expression of p-ERK increased at P9, while there was a reduction of p53 and p21. In contrast, cyclin D1 was unaffected (Fig 2B). The corresponding densitometry analysis is shown in Fig 2B (right panel).

Taken together, we suggest that miR-200c-3p might favor proliferation and stemness by contrasting the p53/p21 axis.

### MiR-200c-3p affects ASC stemness gene expression

**Fig 3:**
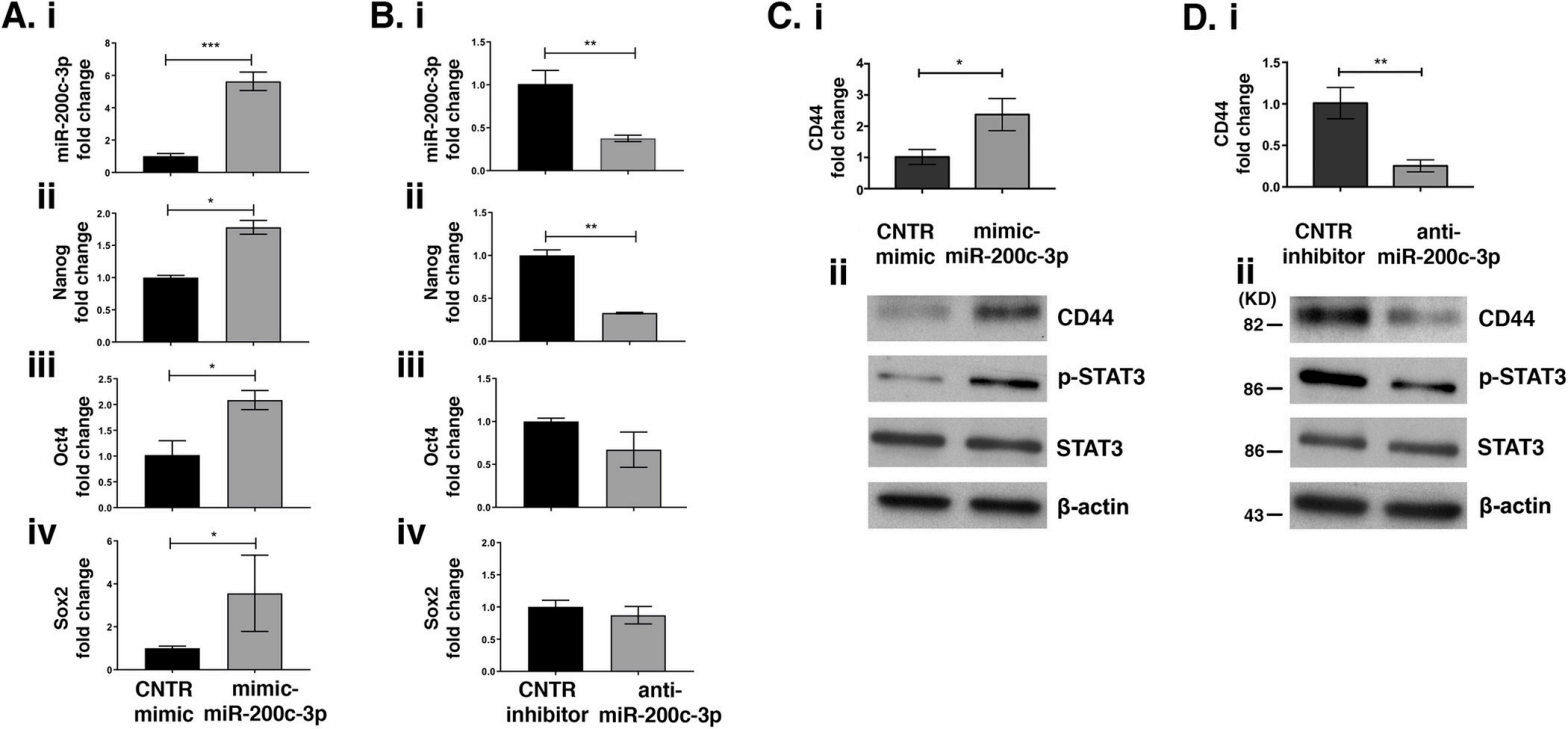
MiR-200c-3p regulates the expression of stemness markers. **A.** qRT-PCR of miR-200c-3p **(i),** Nanog **(ii)**, Oct4 **(iii)** and Sox2 **(iv)** in ASCs at P4 transfected with miR-200c-3p mimic. The fold change of miR-200c-3p was normalized to U6 and that of stemness markers to GAPDH. SD± is the mean of three experiments and the p values were calculated with unpaired t test, *p<0.05, ***p<0.001. **B.** qRT-PCR of miR-200c-3p **(i)** in ASCs transfected with anti-miR-200c-3p. Nanog **(ii)**, Oct4 **(iii)** and Sox2 **(iv)** expressions were normalized to GAPDH. SD± is the mean of three experiments and the p values were calculated with unpaired t test, **p<0.01. **C.** CD44 mRNA was detected by qRT-PCR **(i)** and protein expression of CD44, p-STAT3 and STAT3 was detected by WB **(ii)**, in mimic-miR-200c-3p transfected ASCs. **D.** CD44 mRNA was detected by qRT-PCR **(i)** and protein expression of CD44, p-STAT3 and STAT3 was detected by WB **(ii)**, in anti-miR-200c-3p transfected ASCs. GAPDH was used as housekeeping for qRT-PCR and β-actin in WBs, as a loading control. SD± is the mean of three experiments and the p values were calculated with unpaired t test, *p<0.05, **p<0.01.

To test the hypothesis that miR-200c-3p could regulate stemness of ASCs, we performed mimic-miR-200c-3p transfections in ASCs at P4 when stemness markers expression is low (Fig 1B). At 72 h post-transfection, miR-200c-3p expression was verified by qRT-PCR (Fig 3Ai). The increase in miR-200c-3p was accompanied by a significant increase of Nanog, Oct4 and Sox2 mRNA transcripts (Fig 3Aii–3Aiv). Conversely, inhibition of miR-200c-3p in ASCs, at P9 (Fig 3B), showed a significant reduction of Nanog expression, while Oct4 and Sox2 expressions were not affected (Fig 3Bii–3Biv). These data suggest that miR-200c-3p most likely acts specifically on Nanog. This is consistent with suggestions by other groups that Nanog owns a hierarchical role in regulating pluripotency in ASCs [36]. In addition, strong evidence suggests that CD44, a surface stemness marker, mediates the activation of Nanog [37]. We therefore analyzed CD44 expression at both mRNA and protein levels in ASCs. We observed that mimic miR-200c-3p increased CD44 expression but the inhibitor had the opposite effect. (Fig 3Ci, 3Cii, 3Di and 3Dii). Since Nanog and CD44 expression is often correlated with high p-STAT3, we investigated if miR-200c-3p could influence the expression of these two proteins via p-STAT3. Indeed, over-expression of miR-200c-3p had a positive effect on p-STAT3 expression (Fig 3Ci and 3Cii), whereas inhibition of this miRNA had an opposite effect (Fig 3Di and 3Dii).

### MiR-200c-3p induces proliferation and contrasts senescence of ASCs

**Fig 4:**
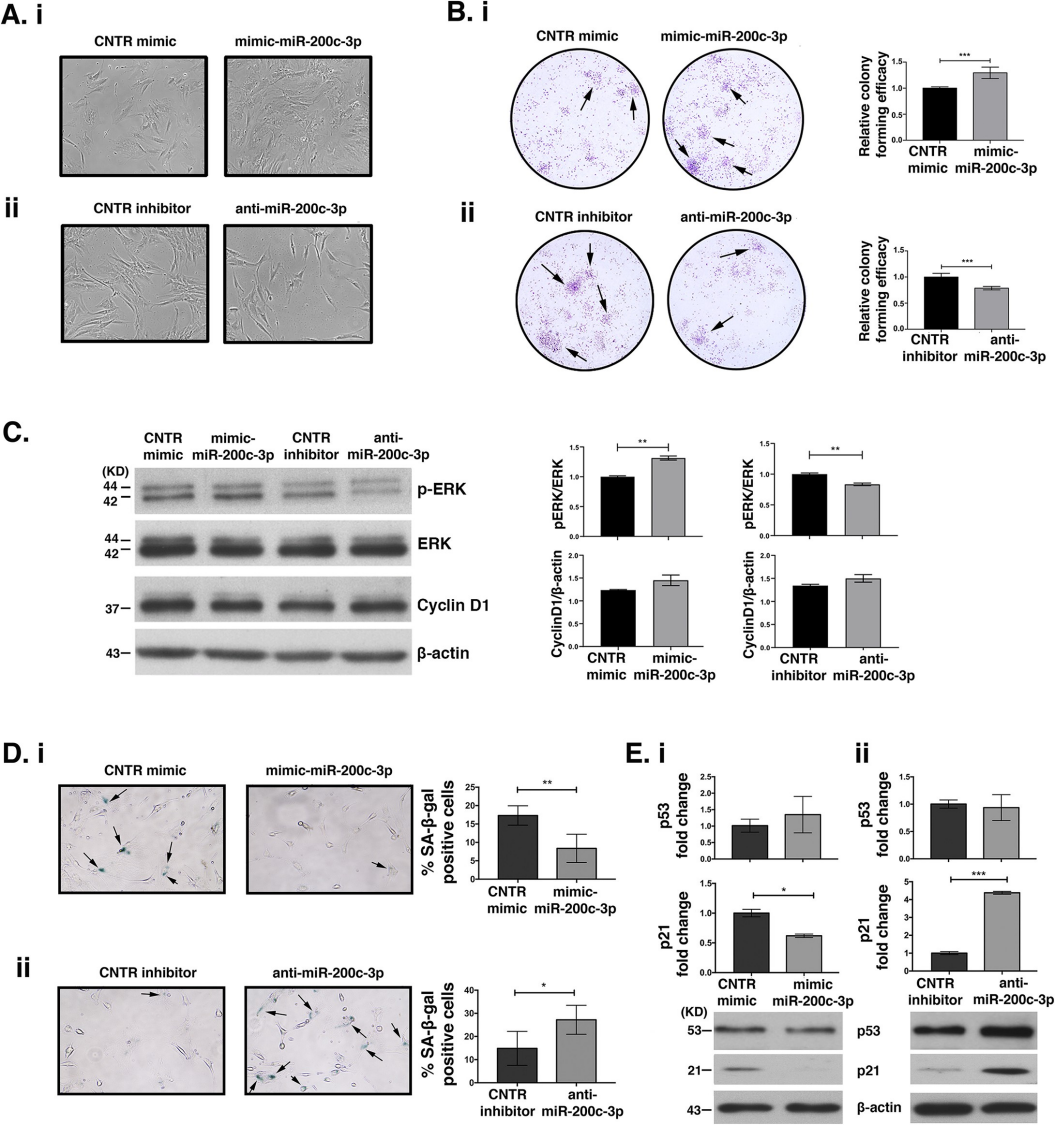
MiR-200c-3p affects proliferation and regulates senescence through p53, p21 expression in ASCs. **A.** Representative images of ASCs transfected with mimic at P4, (low miR-200c-3p) **(i)** or inhibitor miR-200c-3p at P9, (high miR-200c-3p) **(ii)** and their corresponding controls. The images were taken at 72 h post-transfection at 40x magnification. **B.** Clonogenic assays of ASCs transfected with miR-200c-3p mimics **(i)** and anti-miR-200c **(ii)** or their respective controls. Cells were stained with crystal violet and subsequently photographed. Absorbance at 595 nm was measured to estimate the optical density in each condition. The black arrows indicate colonies formation. SD± is the mean of optical density of each condition. The experiment was repeated twice and each condition in triplicates and p values were calculated with unpaired t test, ***p<0.001. **C.** Expression of p-ERK, ERK, Cyclin D1 was studied by WB in ASCs transfected either with mimic at P4 or with anti-miR-200c-3p at P9. On the right, densitometry analysis measures the levels of p-ERK normalized to ERK and Cyclin D1 normalized to β-actin. SD± is the mean of two WBs and the p values were calculated with unpaired t test, **p<0.01. **D. (i):** The percentage of cells positive for SA-β-galactosidase in ASCs transfected with miRNA mimics or **(ii):** inhibitors was calculated by counting blue cells in at least 7–8 different areas and in triplicates for each condition. The images (magnification 20x) are representative of one out of three wells. The black arrows indicate β-gal positive cells. SD± is the mean of the % β-gal positive cells detected in three wells for each condition and the p values were calculated with unpaired t test, *p<0.05, **p<0.01. **E.** Detection of p53, p21 transcripts was performed by qRT-PCR in ASCs transiently transfected at 72 h with **(i):** mimic-miR-200c-3p, at P4, and the corresponding WBs or **(ii):** anti-miR-200c-3p, at P9 and the corresponding WBs. GAPDH was used for gene normalization in qRT-PCR and β-actin, loading control for proteins. SD± is the mean of three independent experiments. P values were calculated with two tailed unpaired t test, *p<0.05, ***p<0.001.

The effects of miR-200c-3p on ASCs proliferation were evaluated through various approaches. First, we noticed an increased confluence in ASCs at P4, (low miR-200c-3p) transfected with mimic miR-200c-3p, at 72 h (Fig 4Ai, upper panels). Conversely, anti-miR-200c-3p transfection at P9, (high miR-200c-3p) negatively affected cell proliferation (Fig 4Aii, lower panels). We also evaluated the ability of ASCs to form colonies at low-density inoculation, which is an index of the proliferation capacity. MiR-200c-3p over-expression increased the number of cells, whereas its inhibition had an opposite effect (Fig 4Bi and 4Bii, respectively). Next, we assessed the activation of ERK phosphorylation (p-ERK) and cyclin D1 expression by WB (Fig 4C). A slight increase of p-ERK expression was noticed when miR-200c-3p was over-expressed, whereas anti-miR-200c-3p had a slight negative effect on p-ERK (Fig 4C, WB and densitometry). Cyclin D1 remained unchanged in mimic/inhibitor-transfected cells. Since proliferation and senescence are intimately connected [38] we investigated the role of miR-200c-3p in ASCs senescence by SA-β-galactosidase staining. The percentage of β-galactosidase positive cells transfected with mimic miR-200c-3p was less than those transfected with anti-miR-200c-3p (Fig 4Di and 4Dii). Since, p53 and p21 expression were modulated during *in vitro* passages of ASCs and both regulate cell senescence [37, 39], and the biological function of p21 is to counteract cell cycle progression genes and to upregulate senescence-inducing genes [40], we looked at the impact of miR-200c-3p on these proteins. In ASCs transfected with mimic-miR-200c-3p at P4 or anti-miR-200c-3p at P9, the levels of p53, p21 transcripts were assessed by qRT-PCR (Fig 4E). Over-expression or inhibition of this miRNA in ASCs had no effect on p53 mRNA (Fig 4Ei and 4Eii upper graphs, respectively). This result is consistent with only a marginal decrease or increase of p53 protein levels in ASCs transfected with either mimics or inhibitors, respectively (Fig 4Ei and 4Eii WBs). P21 mRNA and protein levels were significantly affected by miR-200c-3p over-expression or inhibition (Fig 4Ei and 4Eii, respectively).

Overall, these results suggest a pro-proliferative and anti-senescence role of miR-200c-3p in ASCs via p-ERK and interference with p21-driven cellular senescence.

### MiR-200c-3p regulates PD-L1 and IDO1

**Fig 5:**
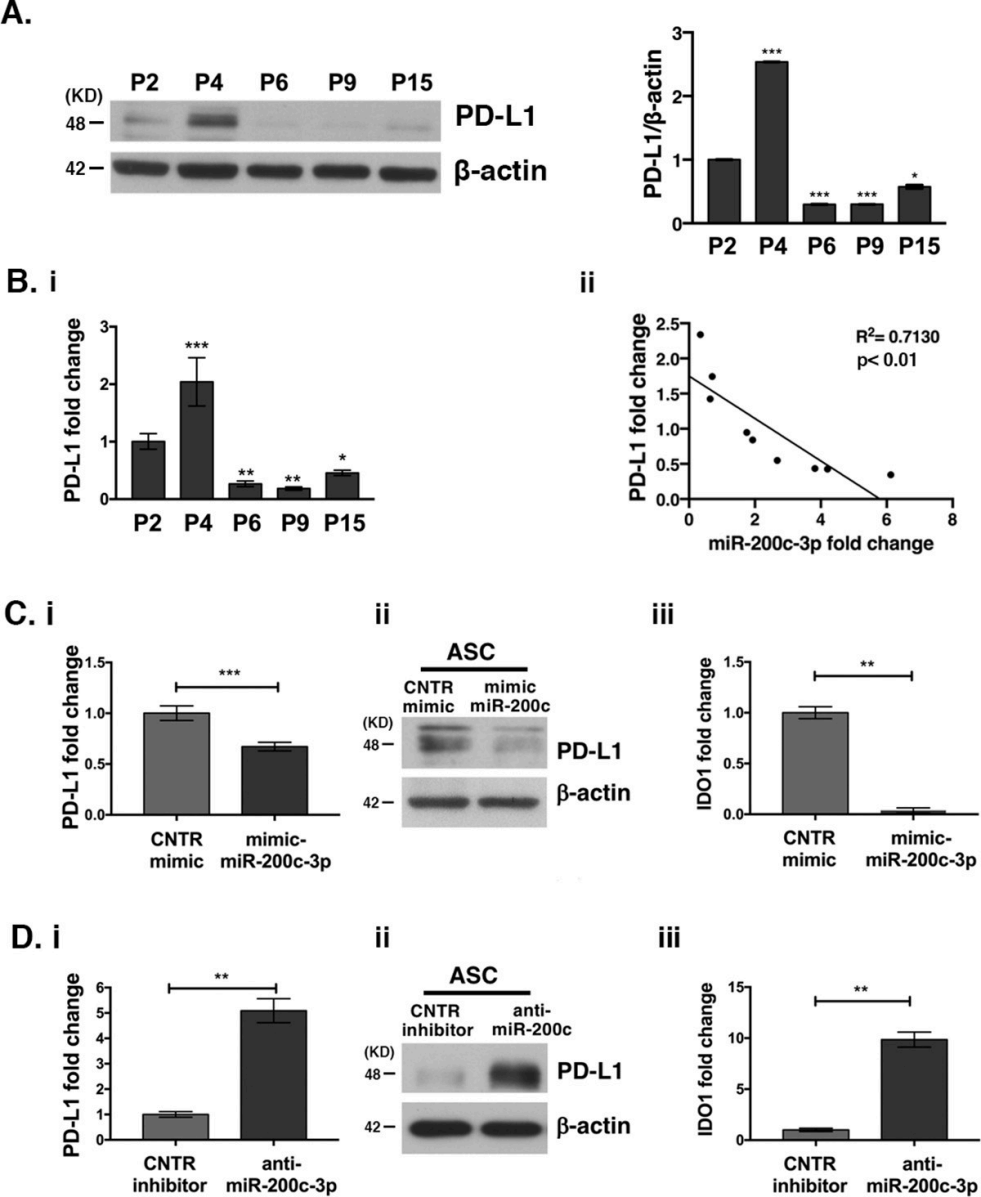
MiR-200c-3p inverse correlates with PD-L1 expression and modulates PD-L1 and IDO1 expression in ASCs. **A.** PD-L1 expression at P2-P15 passages was assessed by WB. Densitometric analysis measures the fold change of PD-L1 expression normalized to β-actin in each passage. One out of two WBs is shown. Dunnett’s multiple comparisons test was applied to compare P2 with each of the passages. *p<0.05, ***p<0.001. **B. (i):** PD-L1 mRNA detection by QRT-PCR, during *in vitro* passages (P). PD-L1 expression was normalized to GAPDH. SD± are the mean of three independent experiments. Dunnett’s multiple comparisons test was applied to compare P2 with each of the passages. *p<0.05, **p<0.01, ***p<0.001. **(ii):** Correlation analysis between PD-L1 and miR-200c-3p expression was performed in ASCs derived from 5 healthy donors at all passages from 2 to 15. The square of the correlation coefficient, R^2^ <0.7 shows that the inverse correlation is significant with p<0.01. **C. (i):** PD-L1 transcript fold change by qRT-PCR in ASCs transfected with miR-200c-3p mimics, at P4 and **(ii):** PD-L1 protein detection by WB. **(iii):** IDO1 transcript fold change by qRT-PCR in ASCs transfected with miR-200c-3p mimics, at P4. **D. (i):** PD-L1 transcript by qRT-PCR in ASCs transfected with miR-200c-3p inhibitors, at P9 and **(ii):** PD-L1 protein detection by WB. and **(iii):** IDO1 transcript fold change by qRT-PCR in ASCs transfected with miR-200c-3p inhibitor, at P9. SD± is the mean of three experiments and the p values were calculated with two tailed unpaired t test. **p<0.01, ***p<0.001.

As mentioned earlier, miR-200c-3p expression varies during *in vitro* passages with the highest picks at P6-P9, accompanied by an increase of the stemness markers Nanog, Oct4 and Sox2 (Fig 1A and 1B). PD-L1 is a validated target of miR-200c-3p [41]. Since there is little information on PD-L1 expression during *in vitro* passages of ASCs, we assessed its expression by WB (Fig 5A). A consistent PD-L1 increase at P4 followed by its reduction at P6 and P9, was observed. This pattern of expression is opposite to that of miR-200-3p (Fig 5A, WB and the corresponding densitometry). PD-L1 mRNA data are consistent with the protein data (Fig 5Bi). Next, we confirmed the negative correlation between PD-L1 and miR-200c-3p expression in ASCs at various passages by correlation analysis (Fig 5Bii). To confirm the inverse correlation between PD-L1 and miR-200c-3p at P4 and P9, ASCs were transfected with mimics at P4 or inhibitors at P9 respectively (Fig 5C and 5D). At P4, over-expression of miR-200c-3p had a negative effect on PD-L1 mRNA and protein (Fig 5Ci and 5Cii). In contrast, at P9, inhibition of miR-200c-3p induced PD-L1 expression both at the transcriptional and at the protein level (Fig 5Di and 5Dii). Similar to PD-L1, IDO1, is known to exert immunosuppressive effects [42]. So, we assessed the levels of IDO1 in ASCs transfected with miR-200c-3p mimics and with miR-200c-3p inhibitors (Fig 5Ciii and 5Diii). The data show that miR-200c-3p inhibition induced IDO1, while overexpression of miR-200c-3p had the opposite effect. These results indicate an immunomodulatory role of miR-200c-3p in ASCs.

## Discussion

ASCs represent a very important tool in regenerative medicine and tissue repair due to their pluripotency. However, a major caveat for their application in the clinic is the loss of stemness and senescence during *in vitro* passage. In this study, we have asked if miRNAs can be employed to regulate these two characteristics in ASCs to broaden their clinical application.

Indeed, a few studies have demonstrated that miRNAs play a critical role in the control of pluripotency-related genes. Among these miRNAs, miR-200c has been successfully used to reprogram mouse or human induced pluripotent stem cells (iPSCs) and human embryonic stem cells (hESC) [23, 43]. For instance, inhibition of miR-200c in hESCs inhibited Nanog expression [28] and consequently self-renewal and promoted differentiation [44]. In contrast, its over-expression induced Nanog expression and decreased apoptosis, resulting in maintenance of self-renewal and proliferation of these cells [45]. Whilst this miRNA seems to play a significant role in hESC self-renewal, its effect of miR-200c-3p in ASCs pluripotency has not been fully explored. In this study, we demonstrated that over-expression of miR-200c-3p favored stemness by inducing Nanog, Oct4 and Sox2 expression. Our results are also consistent with data reported in embryonic stem cells studies in embryonic stem cells [46]. Particularly, the Nanog expression is considered a *bonafide* stemness marker having a hierarchical role in regulating the pluripotency and plasticity of ASCs [36]. In agreement with these data, we also noted that the effect of miR-200c-3p was more pronounced on Nanog than on Oct4 and Sox2, at P6 and P9 passages [47]. In cancer cell lines context, CD44 facilitates translocation of Nanog to the nucleus. The nuclear Nanog, in turn, forms a complex with STAT-3 and activates it. As a consequence, pluripotent stem cell regulators are activated [48]. Similar findings have been reported also in ASCs [37]. Park et al., have shown that CD44/Nanog/p-STAT3 pathway was downregulated by miR-34a, a known anti-proliferative and pro-senescence miRNA, by targeting Nanog [37]. The data presented here suggest that miR-200c-3p positively regulated Nanog. We also found that over-expression of this miRNA induced expression of CD44 and p-STAT3 in ASCs. The role of STAT3 in regulation of Nanog and hence in both mouse ESC and iPSC has been previously demonstrated [49]. How does miR-200c-3p positively regulate these genes is a matter of open debate and future studies. However, it can be surmised that a negative regulator of CD44/p-STAT3 axis could be downregulated by it. It is also conceivable that this miRNA could negatively influence miR-34a, which is known to inhibit the CD44/Nanog/STAT3 axis. Some miRNAs are known to localize in nucleus to negatively regulate maturation of other miRNAs [50]. Most interestingly, it has been observed that miR-200c can migrate to the nucleus [51]. Based on these and our observations, it is tempting to speculate that miR-34a maturation could be affected by miR-200c. Overall, our data suggest that the stimulation of CD44/p-STAT3 axis could account for miR-200c-3p-mediated induction of stemness markers.

MiR-200c-3p overexpression in ASCs increased their confluency and colony forming ability, most likely through activation of p-ERK and concomitant downregulation of p21. In epithelial cancer cells, Carter et al, have shown that increased expression of miR-200 family, induced KRAS expression via binding to the 3’UTR of Ras association domain family member 2 (RASSF2), a negative regulator of KRAS. This led to activation of MAPK/ERK pathway and specifically p-ERK via KRAS [52]. We speculate that akin to the observations by Carter et al, miR-200c-3p might affect similar pathways to upregulate p-ERK.

P53 and p21 has been shown to be closely linked with senescence [35]. This senescence-inducing effect is most likely due to proteasome-dependent degradation of Nanog [53]. We investigated if over-expression of miR-200c-3p and up-regulation of Nanog could adversely affect p53/p21 expression. Our data are consistent with the suggestion that the antisenescence effect of miR-200c-3p is most likely through down-regulation of p21. It remains to be investigated if the marginal reduction in p53 observed by us could be involved in the anti-senescence effect of miR-200c-3p in ASCs.

As far as PD-L1 expression is concerned, little is known about how it is regulated during *in vitro* passages and expansion of ASCs. Our results showed that PD-L1 and miR-200c-3p are differentially expressed in early and late passages of ASCs. That miR-200c-3p inhibits PD-L1 expression was previously demonstrated in Acute Myeloid Leukemia (AML) and in ovarian cancer (OC) [41, 54]. In addition, the pattern of the immunosuppressive enzyme IDO1 followed that of PD-L1. How the regulation of these two proteins by miR-200c-3p affects immunomodulatory properties of ASCs, needs further investigation. A study showed that PD-L1 expression was low in the early passages of ASCs and was induced when ASCs were placed in contact with allogeneic activated T-cells, in a co-culture setting [16]. T-cells were not able to attack and kill high PD-L1-expressing-ASCs, favoring allogeneic ASCs implementation for tissue engineering and therapeutic applications. In our 2D cultures of ASCs, the decrease of PD-L1 expression most likely was due to the increased miR-200c-3p expression. It would be interesting to assess the endogenous levels of miR-200c-3p expression in ASCs when placed with activated T cells in a co-culture setting to test whether there is a back-forward loop between miR-200c-3p and PD-L1.

It cannot be excluded that PD-L1 expression might be higher in later passaged ASCs. In such a scenario, over-expression of miR-200c-3p could be used to moderate PD-L1 expression to fine-tune immunomodulation capacity and enhance self-renewal. It would be also interesting to test different concentrations of miR-200c-3p mimics or inhibitors to modulate PD-L1 expression in early or late passaged ASCs and assess T-cell responses in allogenic co-cultures. Assessment of immune responses might shed light into personalized therapeutic applications.

To our knowledge, this is the first study to provide evidence that miR-200c-3p favors stemness, contrasts senescence and induces proliferation in ASCs. We further show that these characteristics of ASCs are influenced through ERK and p21/p53 pathways as well as by activating the CD44/p-STAT3/ Nanog axis. Our data could have important therapeutic implications in which miR-200c-3p mimics can be used to switch on proliferation and inhibit senescence of ASCs in order to enhance their expansion for use in cell-based clinical approaches. The fact that miR-200c-3p also targets PD-L1 strengthens its candidature as a target for regulating immunomodulation of ASCs and potentiate their therapeutic applications in the context of inflammatory/autoimmune diseases.

## Conclusions

This study provides evidence of miR-200c-3p involvement in self-renewal and immune modulation of human ASCs.

**Fig 6:**
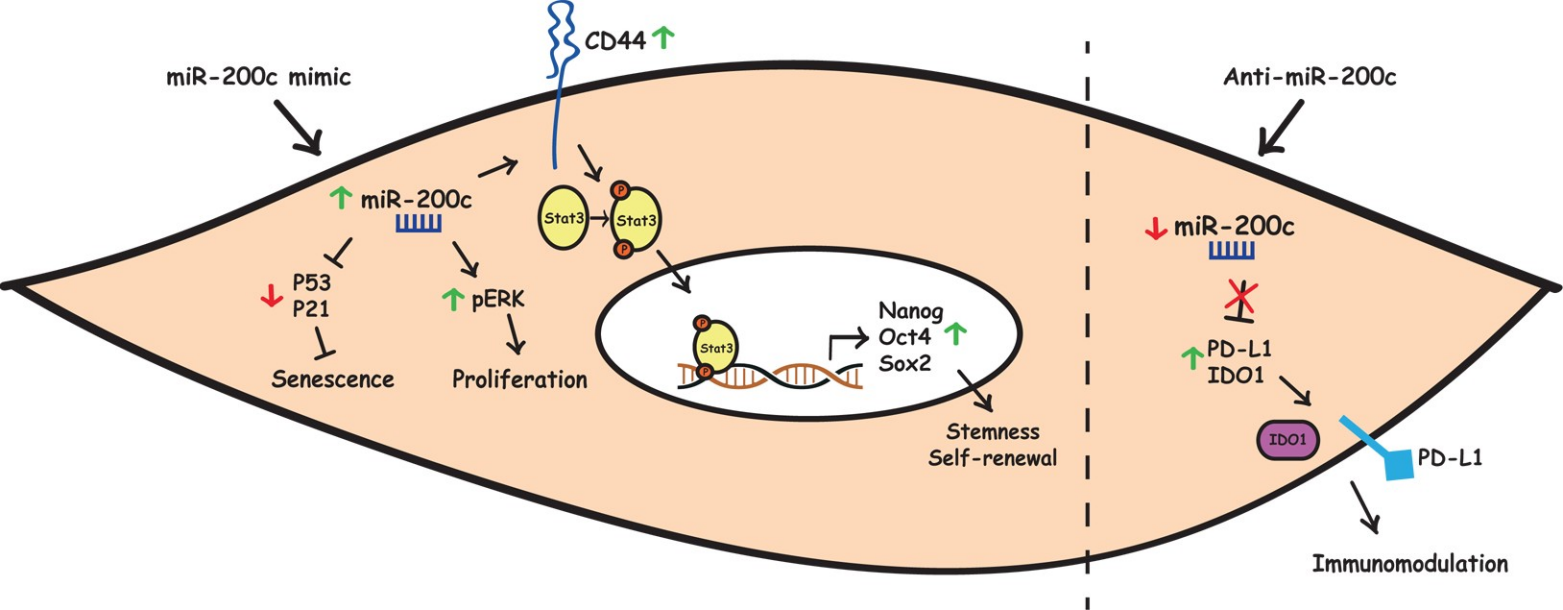
Graphical summary of how miR-200c-3p may regulate self-renewal, proliferation, senescence and have immunomodulatory effect in ASCs. Left: in the cytoplasm, over-expression of miR-200c-3p (green arrow) may stimulate cell proliferation through an increase of p-ERK and inhibit cell senescence by downregulating p53/p21 axis (red arrow). MiR-200c-3p can also increase CD44 expression leading to STAT3 phosphorylation, which in turn may activate transcription of stemness-related genes in the nucleus. Right: Inhibition of miR-200c-3p with a specific anti-miR (red arrow) alleviates repression of PD-L1 and IDO-1, the inhibitory checkpoints, to regulate immune responses against ASCs.

The over-expression of this miRNA by mimic transfection stimulated cell proliferation through increase of p-ERK levels and inhibited cell senescence through downregulation of p53/p21 axis. MiR-200c-3p also increased expression of CD44 receptor and subsequent phosphorylation of STAT3, thus leading to transcriptional stimulation of stemness-related genes like Nanog, Oct4 and Sox2. Conversely, miR-200c-3p inhibition released the expression of PD-L1, a direct target of this miRNA and of the IDO1 enzyme, thus affecting ASC-related immunomodulatory effects (Fig 6). These findings provide new insights into the molecular mechanisms how miR-200c-3p could restore the self-renewal potential of ASCs, thereby potentiating their use in clinical applications.

## Supporting information

S1 Fig Phenotypical characterization of ASCs. Flow cytometric analysis of ASC cells stained with monoclonal antibodies directed against mesenchymal markers (CD29, CD90, CD166, CD44) or hematopoietic markers (CD34, CD45). The isotype-matched monoclonal antibodies (light grey lines) served as a control.
(TIF)

S1 File Supplementary material and methods. (DOCX)

S1 Raw images (PDF)

## References

[1] Ciavarella S, Grisendi G, Dominici M, Tucci M, Brunetti O, Dammacco F, et al. In vitro anti-myeloma activity of TRAIL-expressing adipose-derived mesenchymal stem cells. Br J Haematol. 2012;157(5):586–98. Epub 2012/03/17. doi: 10.1111/j.1365-2141.2012.09082.x . 22420897

[2] Espagnolle N, Hebraud B, Descamps JG, Gadelorge M, Joubert MV, Ferreira LDS, et al. Functional Comparison between Healthy and Multiple Myeloma Adipose Stromal Cells. Stem Cells Int. 2020;2020:4173578. Epub 2020/03/28. doi: 10.1155/2020/4173578; PubMed Central PMCID: PMC7077052 of this article. 32215016 PMC7077052

[3] Herreros MD, Garcia-Olmo D, Guadalajara H, Georgiev-Hristov T, Brandariz L, Garcia-Arranz M. Stem Cell Therapy: A Compassionate Use Program in Perianal Fistula. Stem Cells Int. 2019;2019:6132340. Epub 2019/06/14. doi: 10.1155/2019/6132340; PubMed Central PMCID: PMC6525789. 31191678 PMC6525789

[4] Kim KW, Moon SJ, Park MJ, Kim BM, Kim EK, Lee SH, et al. Optimization of adipose tissue-derived mesenchymal stem cells by rapamycin in a murine model of acute graft-versus-host disease. Stem Cell Res Ther. 2015;6:202. Epub 2015/10/27. doi: 10.1186/s13287-015-0197-8; PubMed Central PMCID: PMC4619057. 26497134 PMC4619057

[5] Ueyama H, Okano T, Orita K, Mamoto K, Sobajima S, Iwaguro H, et al. Local transplantation of adipose-derived stem cells has a significant therapeutic effect in a mouse model of rheumatoid arthritis. Sci Rep. 2020;10(1):3076. Epub 2020/02/23. doi: 10.1038/s41598-020-60041-2; PubMed Central PMCID: PMC7033196. 32080313 PMC7033196

[6] Wagner W, Horn P, Castoldi M, Diehlmann A, Bork S, Saffrich R, et al. Replicative senescence of mesenchymal stem cells: a continuous and organized process. PLoS One. 2008;3(5):e2213. Epub 2008/05/22. doi: 10.1371/journal.pone.0002213; PubMed Central PMCID: PMC2374903. 18493317 PMC2374903

[7] Savickienė J, Baronaitė S, Zentelytė A, Treigytė G, Navakauskienė R. Senescence-Associated Molecular and Epigenetic Alterations in Mesenchymal Stem Cell Cultures from Amniotic Fluid of Normal and Fetus-Affected Pregnancy. Stem Cells Int. 2016;2016:2019498. Epub 2016/11/03. doi: 10.1155/2016/2019498; PubMed Central PMCID: PMC5075644. 27803714 PMC5075644

[8] Pepin ME, Infante T, Benincasa G, Schiano C, Miceli M, Ceccarelli S, et al. Differential DNA Methylation Encodes Proliferation and Senescence Programs in Human Adipose-Derived Mesenchymal Stem Cells. Front Genet. 2020;11:346. Epub 2020/05/01. doi: 10.3389/fgene.2020.00346; PubMed Central PMCID: PMC7174643. 32351540 PMC7174643

[9] Ceccarelli S, Pontecorvi P, Anastasiadou E, Napoli C, Marchese C. Immunomodulatory Effect of Adipose-Derived Stem Cells: The Cutting Edge of Clinical Application. Front Cell Dev Biol. 2020;8:236. Epub 2020/05/05. doi: 10.3389/fcell.2020.00236; PubMed Central PMCID: PMC7180192. 32363193 PMC7180192

[10] Teklemariam T, Purandare B, Zhao L, Hantash BM. Inhibition of DNA methylation enhances HLA-G expression in human mesenchymal stem cells. Biochem Biophys Res Commun. 2014;452(3):753–9. Epub 2014/09/11. doi: 10.1016/j.bbrc.2014.08.152 . 25204503

[11] Schiano C, Benincasa G, Infante T, Franzese M, Castaldo R, Fiorito C, et al. Integrated analysis of DNA methylation profile of HLA-G gene and imaging in coronary heart disease: Pilot study. PLoS One. 2020;15(8):e0236951. Epub 2020/08/14. doi: 10.1371/journal.pone.0236951; PubMed Central PMCID: PMC7425923. 32790754 PMC7425923

[12] Sommese L, Benincasa G, Schiano C, Marfella R, Grimaldi V, Sorriento A, et al. Genetic and epigenetic-sensitive regulatory network in immune response: a putative link between HLA-G and diabetes. Expert Rev Endocrinol Metab. 2019;14(4):233–41. Epub 2019/05/28. doi: 10.1080/17446651.2019.1620103 . 31131681

[13] Sommese L, Paolillo R, Cacciatore F, Grimaldi V, Sabia C, Esposito A, et al. HLA-G and anti-HCV in patients on the waiting list for kidney transplantation. Adv Med Sci. 2018;63(2):317–22. Epub 2018/07/18. doi: 10.1016/j.advms.2018.04.004 . 30015095

[14] Patsoukis N, Wang Q, Strauss L, Boussiotis VA. Revisiting the PD-1 pathway. Sci Adv. 2020;6(38):eabd2712. doi: 10.1126/sciadv.abd2712. 32948597 PMC7500922

[15] Francisco LM, Sage PT, Sharpe AH. The PD-1 pathway in tolerance and autoimmunity. Immunol Rev. 2010;236:219–42. Epub 2010/07/20. doi: 10.1111/j.1600-065X.2010.00923.x ; PubMed Central PMCID: PMC2919275. 20636820 PMC2919275

[16] Zhou K, Guo S, Tong S, Sun Q, Li F, Zhang X, et al. Immunosuppression of Human Adipose-Derived Stem Cells on T Cell Subsets via the Reduction of NF-kappaB Activation Mediated by PD-L1/PD-1 and Gal-9/TIM-3 Pathways. Stem Cells Dev. 2018;27(17):1191–202. Epub 2018/07/07. doi: 10.1089/scd.2018.0033 . 29978730

[17] Mbongue JC, Nicholas DA, Torrez TW, Kim N-S, Firek AF, Langridge WHR. The Role of Indoleamine 2, 3-Dioxygenase in Immune Suppression and Autoimmunity. Vaccines (Basel). 2015;3(3):703–29. Epub 2015/09/18. doi: 10.3390/vaccines3030703 ; PubMed Central PMCID: PMC4586474. 26378585 PMC4586474

[18] Zheng G, Qiu G, Ge M, He J, Huang L, Chen P, et al. Human adipose-derived mesenchymal stem cells alleviate obliterative bronchiolitis in a murine model via IDO. Respir Res. 2017;18(1):119. Epub 2017/06/18. doi: 10.1186/s12931-017-0599-5; PubMed Central PMCID: PMC5472885. 28619045 PMC5472885

[19] Yang HM, Sung JH, Choi YS, Lee HJ, Roh CR, Kim J, et al. Enhancement of the immunosuppressive effect of human adipose tissue-derived mesenchymal stromal cells through HLA-G1 expression. Cytotherapy. 2012;14(1):70–9. Epub 2011/10/01. doi: 10.3109/14653249.2011.613926 . 21954834

[20] Gieseke F, Böhringer J, Bussolari R, Dominici M, Handgretinger R, Müller I. Human multipotent mesenchymal stromal cells use galectin-1 to inhibit immune effector cells. Blood. 2010;116(19):3770–9. Epub 2010/07/21. doi: 10.1182/blood-2010-02-270777 . 20644118

[21] Najar M, Raicevic G, Id Boufker H, Stamatopoulos B, De Bruyn C, Meuleman N, et al. Modulated expression of adhesion molecules and galectin-1: role during mesenchymal stromal cell immunoregulatory functions. Exp Hematol. 2010;38(10):922–32. Epub 2010/06/24. doi: 10.1016/j.exphem.2010.05.007 . 20570633

[22] Krol J, Loedige I, Filipowicz W. The widespread regulation of microRNA biogenesis, function and decay. Nat Rev Genet. 2010;11(9):597–610. Epub 2010/07/28. doi: 10.1038/nrg2843 . 20661255

[23] Balzano F, Cruciani S, Basoli V, Santaniello S, Facchin F, Ventura C, et al. MiR200 and miR302: Two Big Families Influencing Stem Cell Behavior. Molecules (Basel, Switzerland). 2018;23(2):282. doi: 10.3390/molecules23020282. 29385685 PMC6017081

[24] Morin RD, O’Connor MD, Griffith M, Kuchenbauer F, Delaney A, Prabhu AL, et al. Application of massively parallel sequencing to microRNA profiling and discovery in human embryonic stem cells. Genome Res. 2008;18(4):610–21. Epub 2008/02/21. doi: 10.1101/gr.7179508 ; PubMed Central PMCID: PMC2279248. 18285502 PMC2279248

[25] Xu N, Papagiannakopoulos T, Pan G, Thomson JA, Kosik KS. MicroRNA-145 regulates OCT4, SOX2, and KLF4 and represses pluripotency in human embryonic stem cells. Cell. 2009;137(4):647–58. Epub 2009/05/05. doi: 10.1016/j.cell.2009.02.038 . 19409607

[26] Park SM, Gaur AB, Lengyel E, Peter ME. The miR-200 family determines the epithelial phenotype of cancer cells by targeting the E-cadherin repressors ZEB1 and ZEB2. Genes Dev. 2008;22(7):894–907. Epub 2008/04/03. doi: 10.1101/gad.1640608 ; PubMed Central PMCID: PMC2279201. 18381893 PMC2279201

[27] Pan Q, Meng L, Ye J, Wei X, Shang Y, Tian Y, et al. Transcriptional repression of miR-200 family members by Nanog in colon cancer cells induces epithelial-mesenchymal transition (EMT). Cancer Lett. 2017;392:26–38. Epub 2017/02/07. doi: 10.1016/j.canlet.2017.01.039 . 28163188

[28] Huang HN, Chen SY, Hwang SM, Yu CC, Su MW, Mai W, et al. miR-200c and GATA binding protein 4 regulate human embryonic stem cell renewal and differentiation. Stem Cell Res. 2014;12(2):338–53. Epub 2013/12/25. doi: 10.1016/j.scr.2013.11.009 . 24365599

[29] Ceccarelli S, Nodale C, Vescarelli E, Pontecorvi P, Manganelli V, Casella G, et al. Neuropilin 1 Mediates Keratinocyte Growth Factor Signaling in Adipose-Derived Stem Cells: Potential Involvement in Adipogenesis. Stem Cells Int. 2018;2018:1075156. Epub 2018/03/15. doi: 10.1155/2018/1075156; PubMed Central PMCID: PMC5845512. 29535768 PMC5845512

[30] Anastasiadou E, Stroopinsky D, Alimperti S, Jiao AL, Pyzer AR, Cippitelli C, et al. Epstein−Barr virus-encoded EBNA2 alters immune checkpoint PD-L1 expression by downregulating miR-34a in B-cell lymphomas. Leukemia. 2019;33(1):132–47. doi: 10.1038/s41375-018-0178-x 29946193 PMC6327052

[31] Camero S, Camicia L, Marampon F, Ceccarelli S, Shukla R, Mannarino O, et al. BET inhibition therapy counteracts cancer cell survival, clonogenic potential and radioresistance mechanisms in rhabdomyosarcoma cells. Cancer Lett. 2020;479:71–88. Epub 2020/03/23. doi: 10.1016/j.canlet.2020.03.011 . 32200036

[32] Vescarelli E, Gerini G, Megiorni F, Anastasiadou E, Pontecorvi P, Solito L, et al. MiR-200c sensitizes Olaparib-resistant ovarian cancer cells by targeting Neuropilin 1. J Exp Clin Cancer Res. 2020;39(1):3. Epub 2020/01/04. doi: 10.1186/s13046-019-1490-7; PubMed Central PMCID: PMC6939329. 31898520 PMC6939329

[33] Cheng H, Qiu L, Ma J, Zhang H, Cheng M, Li W, et al. Replicative senescence of human bone marrow and umbilical cord derived mesenchymal stem cells and their differentiation to adipocytes and osteoblasts. Mol Biol Rep. 2011;38(8):5161–8. Epub 2010/12/29. doi: 10.1007/s11033-010-0665-2 . 21188535

[34] Ren S, Chen J, Duscher D, Liu Y, Guo G, Kang Y, et al. Microvesicles from human adipose stem cells promote wound healing by optimizing cellular functions via AKT and ERK signaling pathways. Stem Cell Research & Therapy. 2019;10(1):47. doi: 10.1186/s13287-019-1152-x 30704535 PMC6357421

[35] Jain AK, Allton K, Iacovino M, Mahen E, Milczarek RJ, Zwaka TP, et al. p53 regulates cell cycle and microRNAs to promote differentiation of human embryonic stem cells. PLoS Biol. 2012;10(2):e1001268. Epub 2012/03/06. doi: 10.1371/journal.pbio.1001268; PubMed Central PMCID: PMC3289600. 22389628 PMC3289600

[36] Pitrone M, Pizzolanti G, Tomasello L, Coppola A, Morini L, Pantuso G, et al. NANOG Plays a Hierarchical Role in the Transcription Network Regulating the Pluripotency and Plasticity of Adipose Tissue-Derived Stem Cells. Int J Mol Sci. 2017;18(6). Epub 2017/05/27. doi: 10.3390/ijms18061107; PubMed Central PMCID: PMC5485931. 28545230 PMC5485931

[37] Park H, Park H, Pak HJ, Yang DY, Kim YH, Choi WJ, et al. miR-34a inhibits differentiation of human adipose tissue-derived stem cells by regulating cell cycle and senescence induction. Differentiation. 2015;90(4–5):91–100. Epub 2015/12/19. doi: 10.1016/j.diff.2015.10.010 . 26677981

[38] Truong NC, Bui KH, Van Pham P. Characterization of Senescence of Human Adipose-Derived Stem Cells After Long-Term Expansion. Adv Exp Med Biol. 2019;1084:109–28. Epub 2018/09/23. doi: 10.1007/5584_2018_235 . 30242785

[39] Mokhberian N, Bolandi Z, Eftekhary M, Hashemi SM, Jajarmi V, Sharifi K, et al. Inhibition of miR-34a reduces cellular senescence in human adipose tissue-derived mesenchymal stem cells through the activation of SIRT1. Life Sci. 2020;257:118055. Epub 2020/07/08. doi: 10.1016/j.lfs.2020.118055. 32634429

[40] Solozobova V, Blattner C. p53 in stem cells. World J Biol Chem. 2011;2(9):202–14. Epub 2011/09/29. doi: 10.4331/wjbc.v2.i9.202 ; PubMed Central PMCID: PMC3178757. 21949570 PMC3178757

[41] Pyzer AR, Stroopinsky D, Rosenblatt J, Anastasiadou E, Rajabi H, Washington A, et al. MUC1 inhibition leads to decrease in PD-L1 levels via upregulation of miRNAs. Leukemia. 2017;31(12):2780–90. Epub 2017/05/31. doi: 10.1038/leu.2017.163 ; PubMed Central PMCID: PMC5791150. 28555079 PMC5791150

[42] Zhang ML, Kem M, Mooradian MJ, Eliane JP, Huynh TG, Iafrate AJ, et al. Differential expression of PD-L1 and IDO1 in association with the immune microenvironment in resected lung adenocarcinomas. Mod Pathol. 2019;32(4):511–23. Epub 2018/10/28. doi: 10.1038/s41379-018-0160-1 . 30367104

[43] Miyoshi N, Ishii H, Nagano H, Haraguchi N, Dewi DL, Kano Y, et al. Reprogramming of mouse and human cells to pluripotency using mature microRNAs. Cell Stem Cell. 2011;8(6):633–8. Epub 2011/05/31. doi: 10.1016/j.stem.2011.05.001 . 21620789

[44] Kim Y, Kim N, Park SW, Kim H, Park HJ, Han YM. Lineage-specific Expression of miR-200 Family in Human Embryonic Stem Cells during In Vitro Differentiation. Int J Stem Cells. 2017;10(1):28–37. Epub 2017/05/24. doi: 10.15283/ijsc17013 ; PubMed Central PMCID: PMC5488774. 28531914 PMC5488774

[45] Mashayekhi P, Noruzinia M, Zeinali S, Khodaverdi S. Endometriotic Mesenchymal Stem Cells Epigenetic Pathogenesis: Deregulation of miR-200b, miR-145, and let7b in A Functional Imbalanced Epigenetic Disease. Cell J. 2019;21(2):179–85. Epub 2019/03/03. doi: 10.22074/cellj.2019.5903 ; PubMed Central PMCID: PMC6397607. 30825291 PMC6397607

[46] Boyer LA, Lee TI, Cole MF, Johnstone SE, Levine SS, Zucker JP, et al. Core transcriptional regulatory circuitry in human embryonic stem cells. Cell. 2005;122(6):947–56. Epub 2005/09/13. doi: 10.1016/j.cell.2005.08.020 ; PubMed Central PMCID: PMC3006442. 16153702 PMC3006442

[47] Pierantozzi E, Gava B, Manini I, Roviello F, Marotta G, Chiavarelli M, et al. Pluripotency regulators in human mesenchymal stem cells: expression of NANOG but not of OCT-4 and SOX-2. Stem Cells Dev. 2011;20(5):915–23. Epub 2010/10/01. doi: 10.1089/scd.2010.0353 . 20879854

[48] Bourguignon LY, Peyrollier K, Xia W, Gilad E. Hyaluronan-CD44 interaction activates stem cell marker Nanog, Stat-3-mediated MDR1 gene expression, and ankyrin-regulated multidrug efflux in breast and ovarian tumor cells. J Biol Chem. 2008;283(25):17635–51. Epub 2008/04/29. doi: 10.1074/jbc.M800109200 ; PubMed Central PMCID: PMC2427357. 18441325 PMC2427357

[49] Do DV, Ueda J, Messerschmidt DM, Lorthongpanich C, Zhou Y, Feng B, et al. A genetic and developmental pathway from STAT3 to the OCT4-NANOG circuit is essential for maintenance of ICM lineages in vivo. Genes Dev. 2013;27(12):1378–90. Epub 2013/06/22. doi: 10.1101/gad.221176.113 ; PubMed Central PMCID: PMC3701193. 23788624 PMC3701193

[50] Tang R, Li L, Zhu D, Hou D, Cao T, Gu H, et al. Mouse miRNA-709 directly regulates miRNA-15a/16-1 biogenesis at the posttranscriptional level in the nucleus: evidence for a microRNA hierarchy system. Cell Research. 2012;22(3):504–15. doi: 10.1038/cr.2011.137 21862971 PMC3292299

[51] Park CW, Zeng Y, Zhang X, Subramanian S, Steer CJ. Mature microRNAs identified in highly purified nuclei from HCT116 colon cancer cells. RNA Biol. 2010;7(5):606–14. Epub 2010/09/25. doi: 10.4161/rna.7.5.13215 ; PubMed Central PMCID: PMC3073257. 20864815 PMC3073257

[52] Carter JV, O’Brien SJ, Burton JF, Oxford BG, Stephen V, Hallion J, et al. The microRNA-200 family acts as an oncogene in colorectal cancer by inhibiting the tumor suppressor RASSF2. Oncol Lett. 2019;18(4):3994–4007. Epub 2019/10/01. doi: 10.3892/ol.2019.10753 ; PubMed Central PMCID: PMC6759516. 31565080 PMC6759516

[53] Sato A, Okada M, Shibuya K, Watanabe E, Seino S, Suzuki K, et al. Resveratrol promotes proteasome-dependent degradation of Nanog via p53 activation and induces differentiation of glioma stem cells. Stem Cell Research. 2013;11(1):601–10. doi: 10.1016/j.scr.2013.04.004 23651583

[54] Anastasiadou E, Messina E, Sanavia T, Mundo L, Farinella F, Lazzi S, et al. MiR-200c-3p Contrasts PD-L1 Induction by Combinatorial Therapies and Slows Proliferation of Epithelial Ovarian Cancer through Downregulation of β-Catenin and c-Myc. Cells. 2021;10(3). Epub 2021/04/04. doi: 10.3390/cells10030519; PubMed Central PMCID: PMC7998372. 33804458 PMC7998372

