# Supplementary figures and images for: MiR-200c-3p maintains stemness and proliferative potential in adipose-derived stem cells by counteracting senescence mechanisms
Source: PLoS One. 2021 Sep 17;16(9):e0257070. doi: 10.1371/journal.pone.0257070 (PMC8448302; doi:10.1371/journal.pone.0257070)

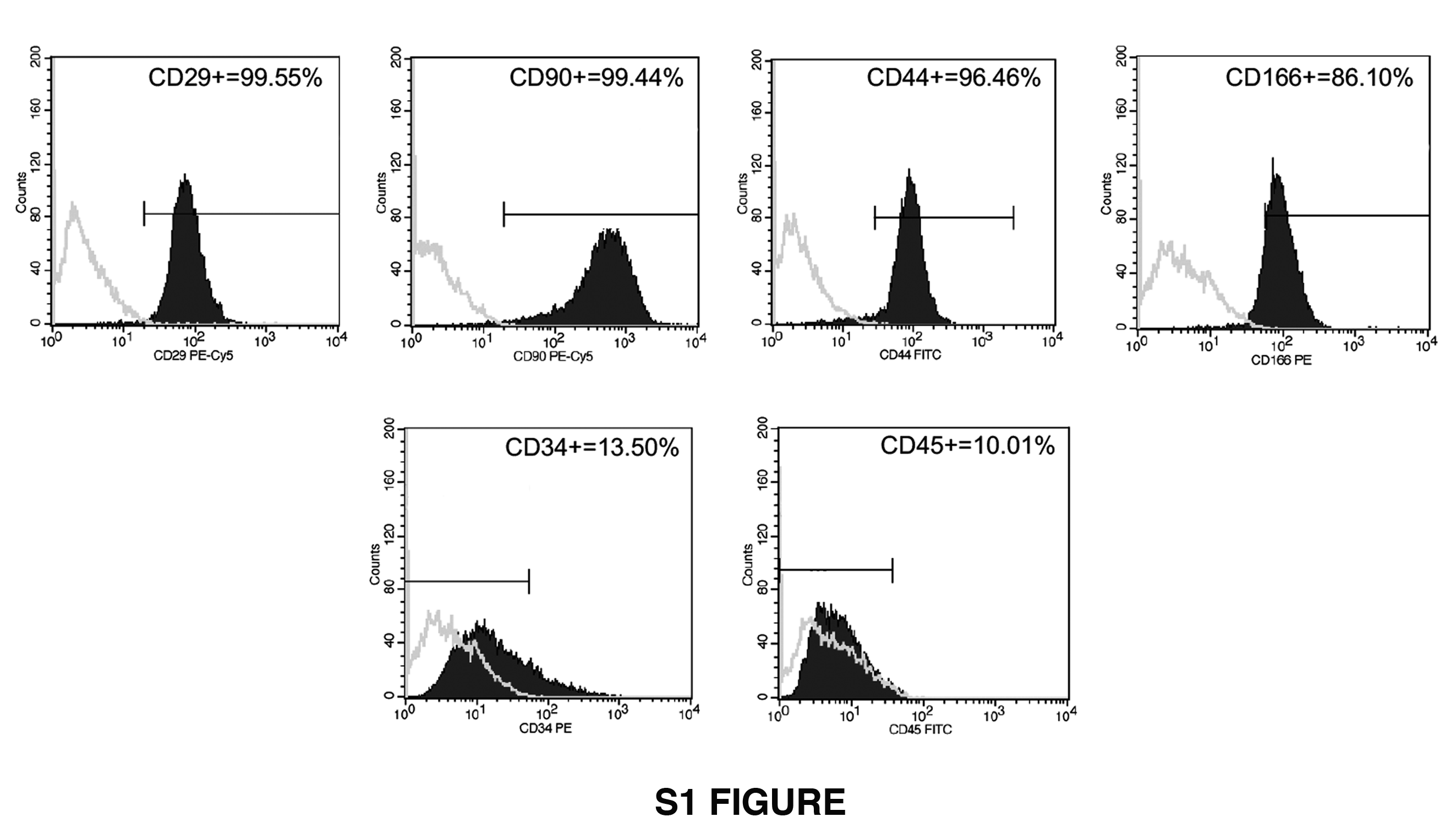

Supplement: S1 Fig — Flow cytometric analysis of ASC cells stained with monoclonal antibodies directed against mesenchymal markers (CD29, CD90, CD166, CD44) or hematopoietic markers (CD34, CD45). The isotype-matched monoclonal antibodies (light grey lines) served as a control. (TIF) [file pone.0257070.s001.tif]

**Figure 2**

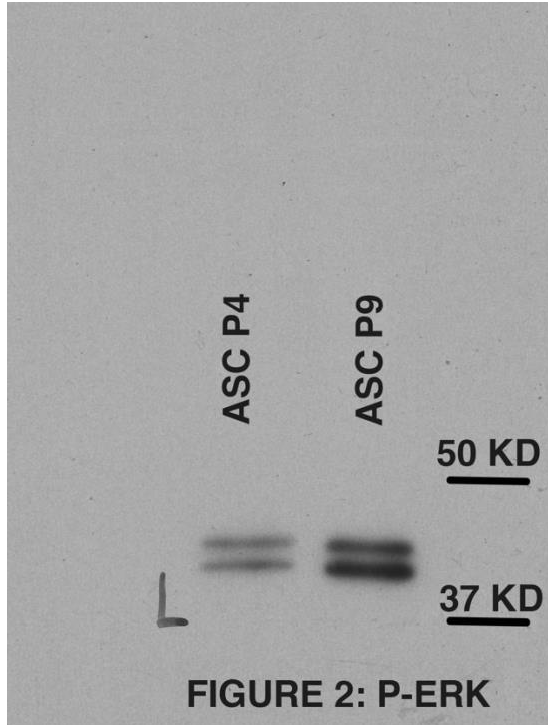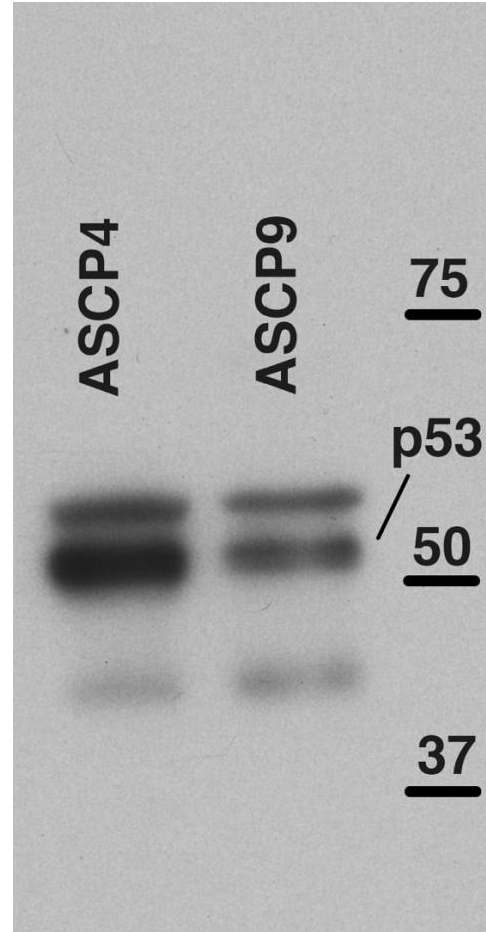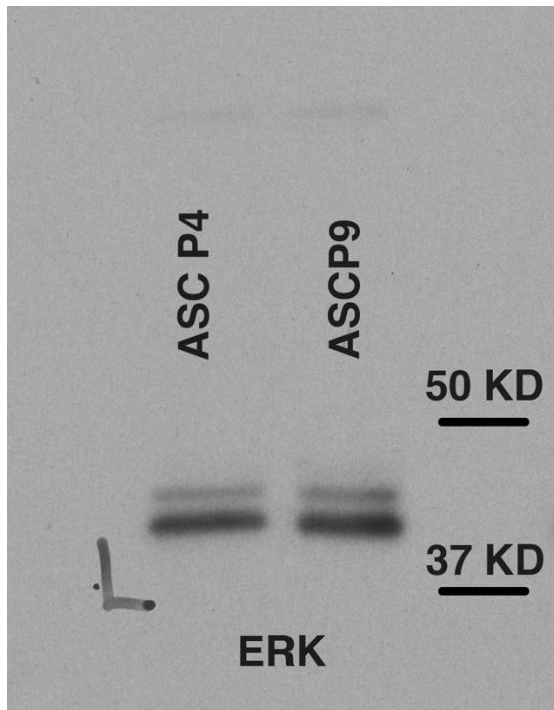

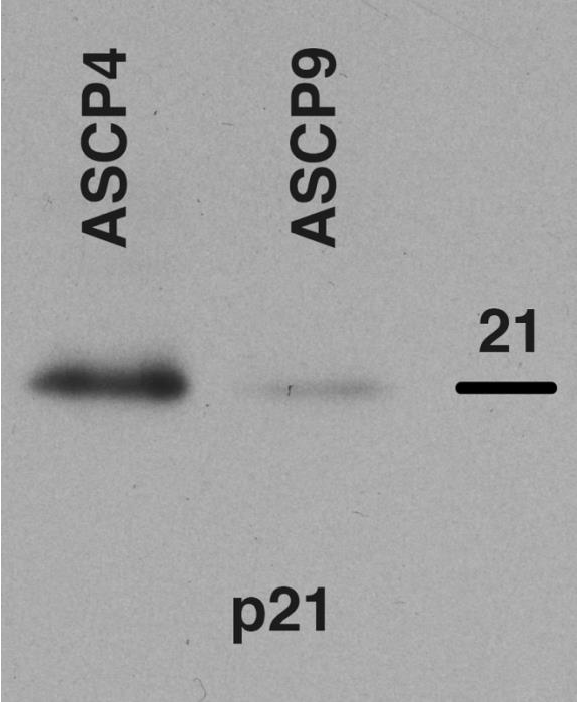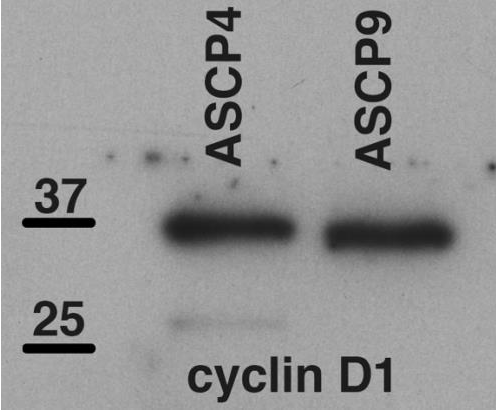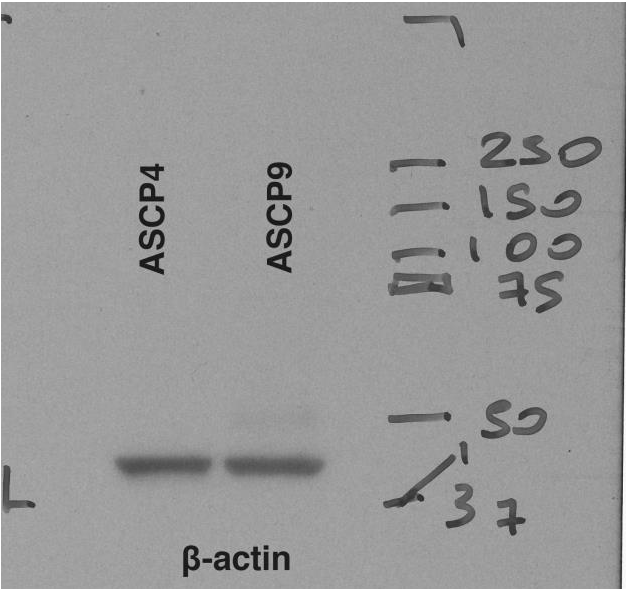

**FIGURE 3**

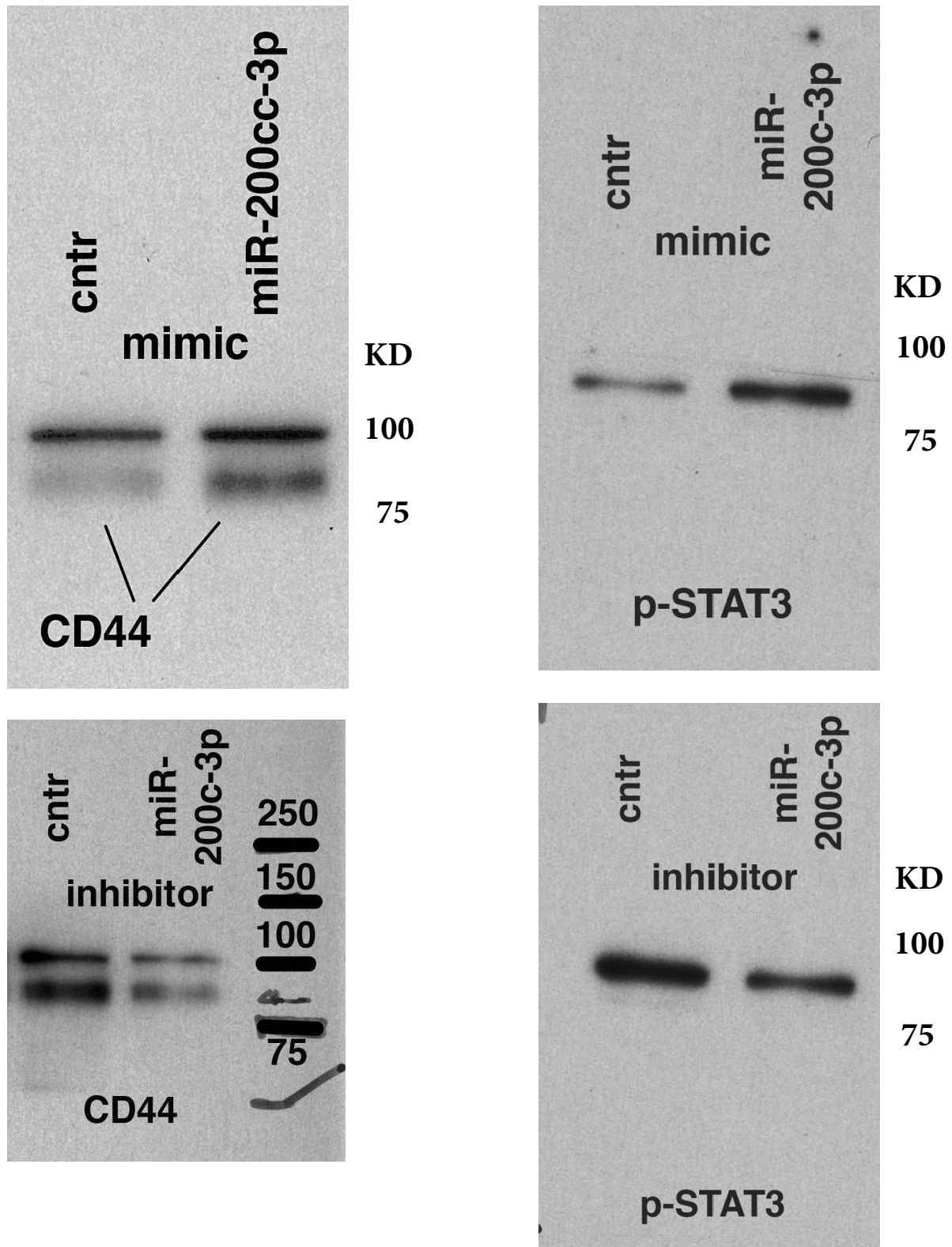

FIGURE 3

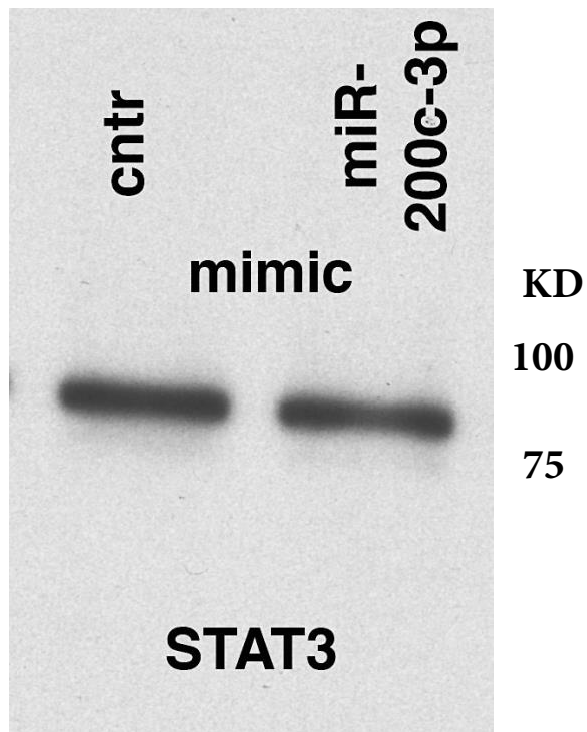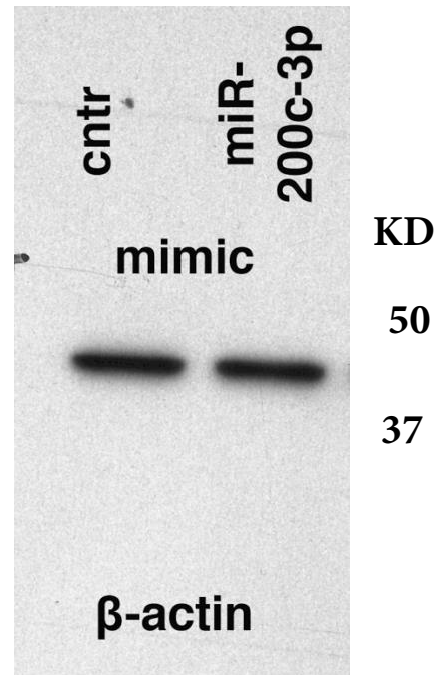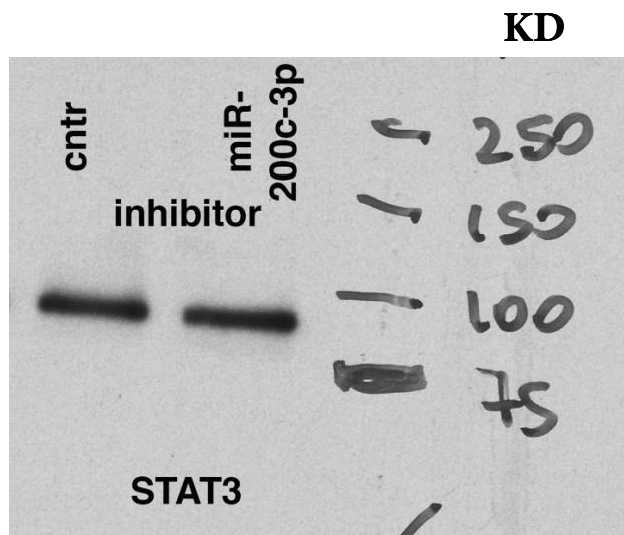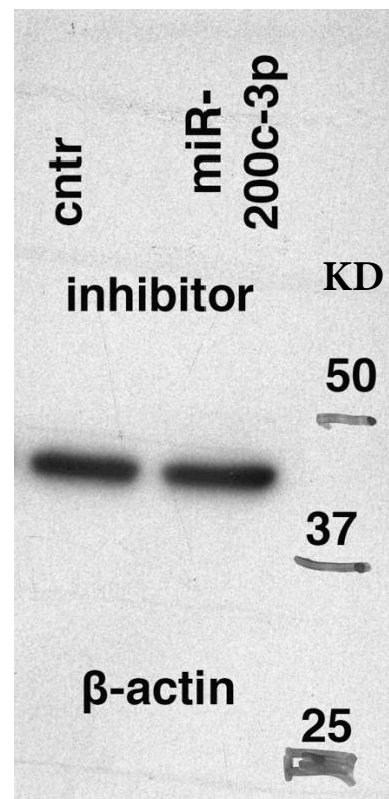

# FIGURE 4

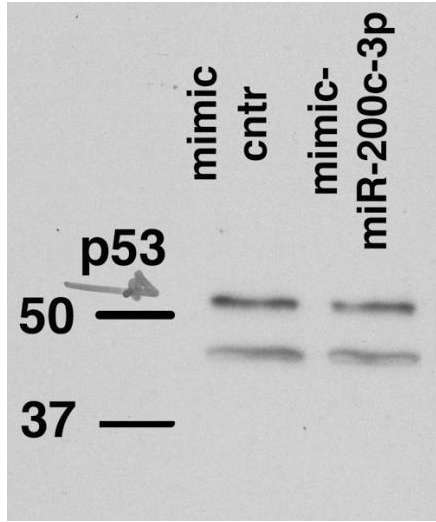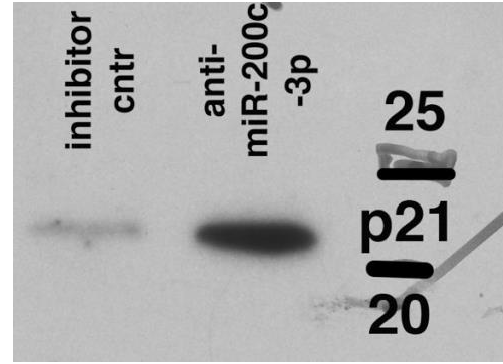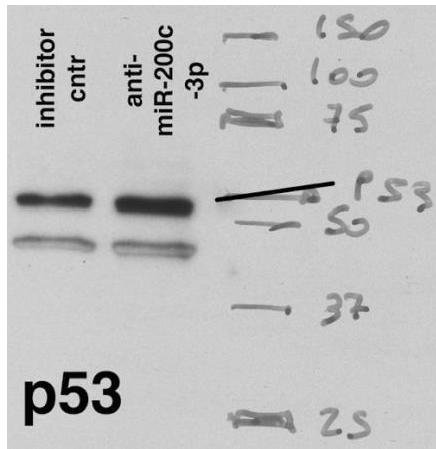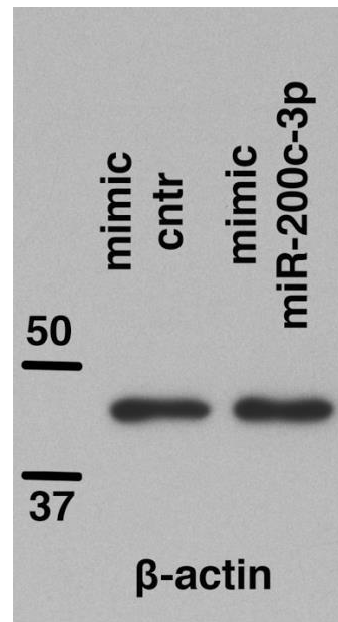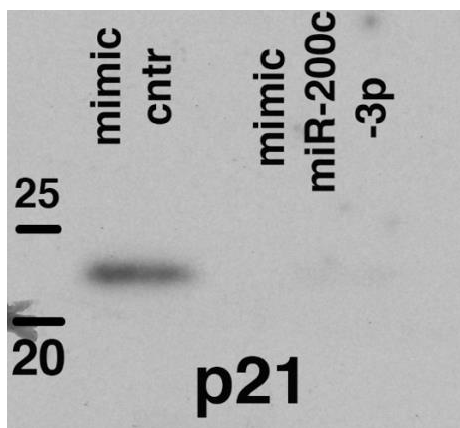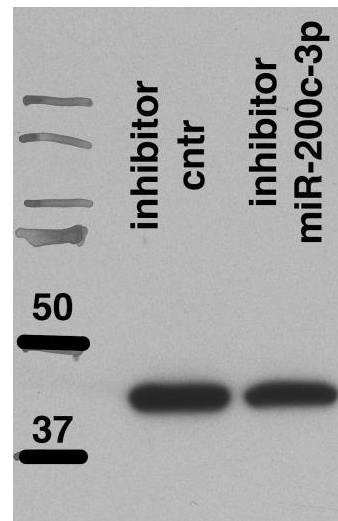

# FIGURE 4

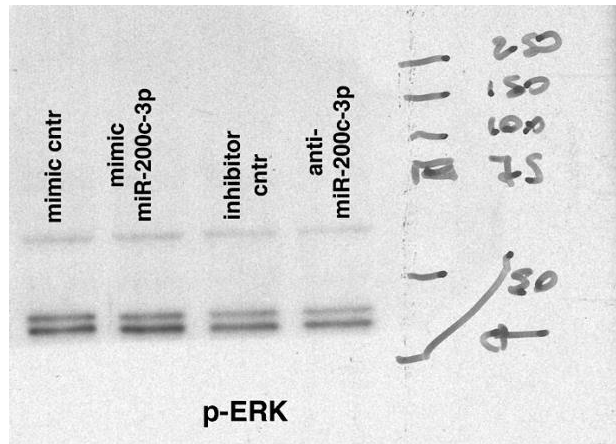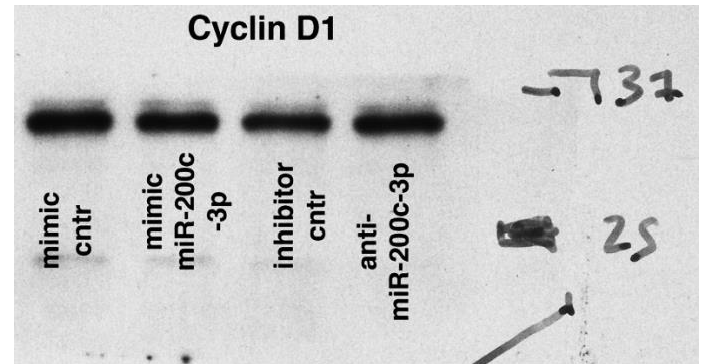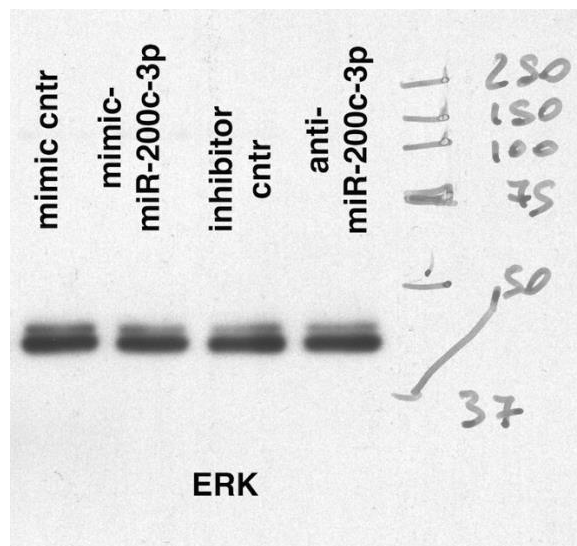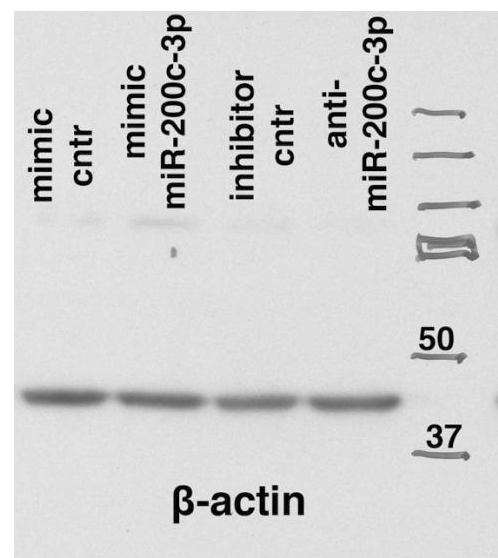

**FIGURE 5**

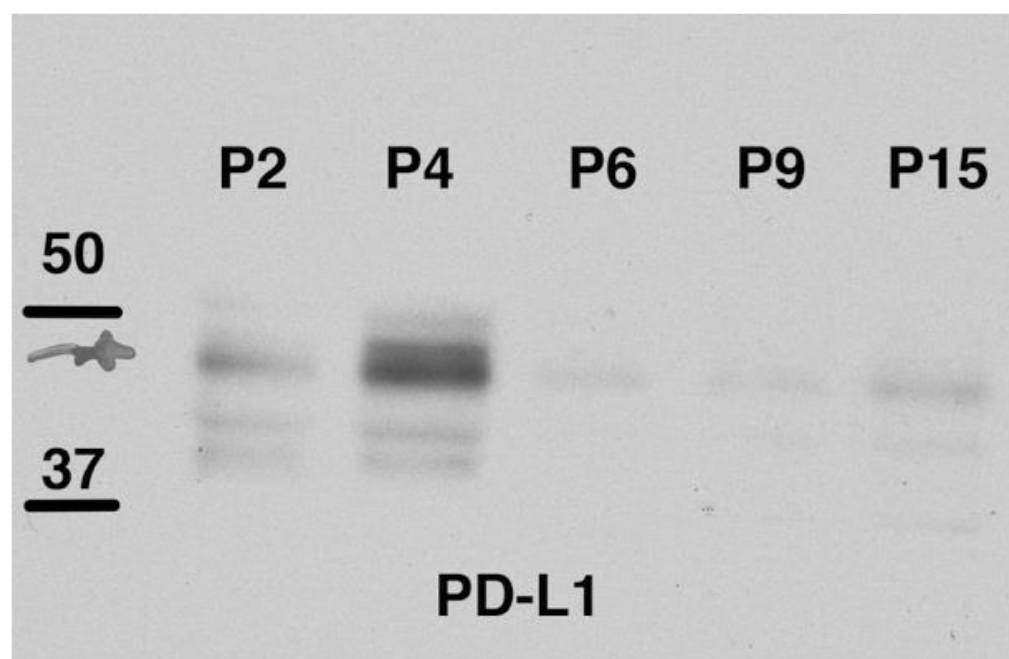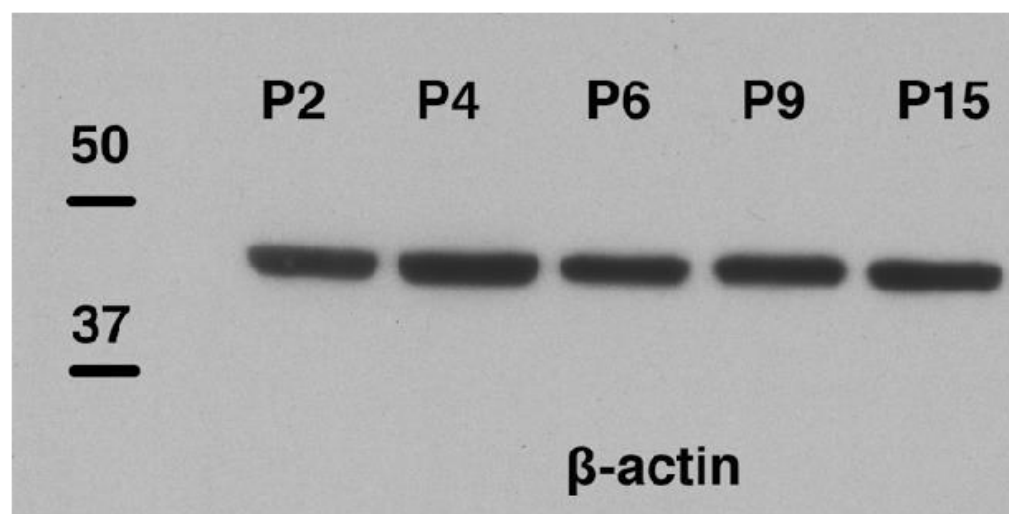

**FIGURE 5**

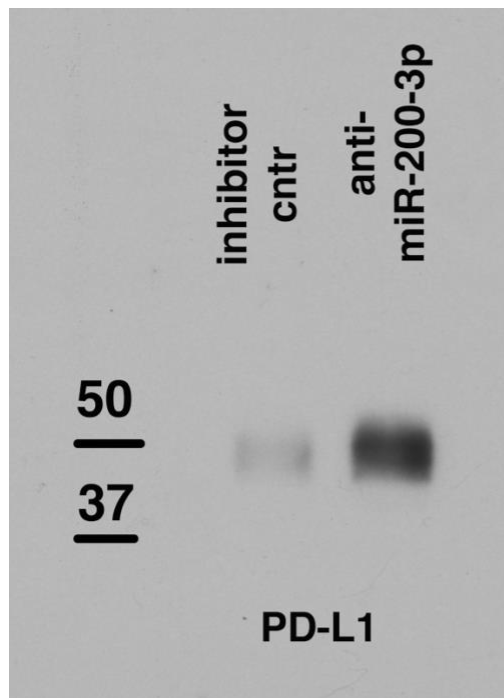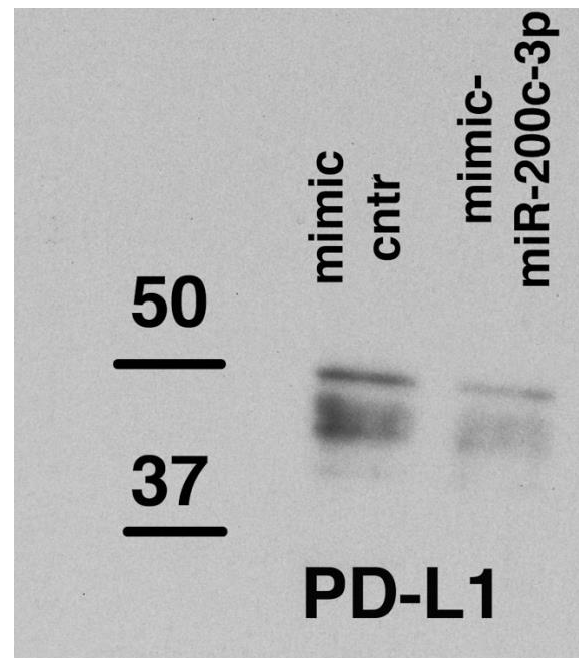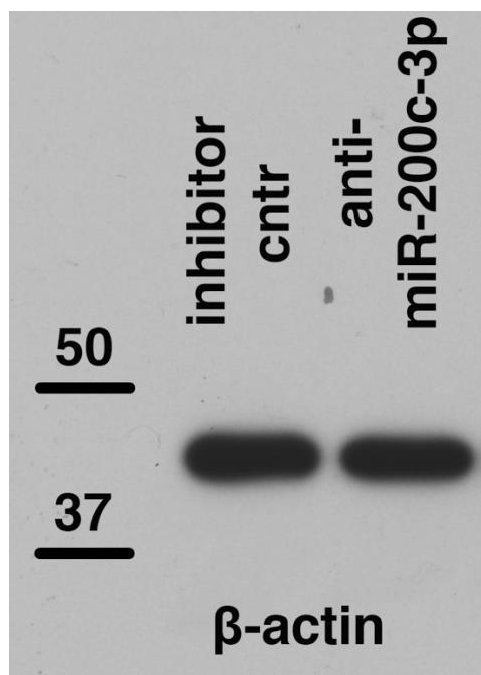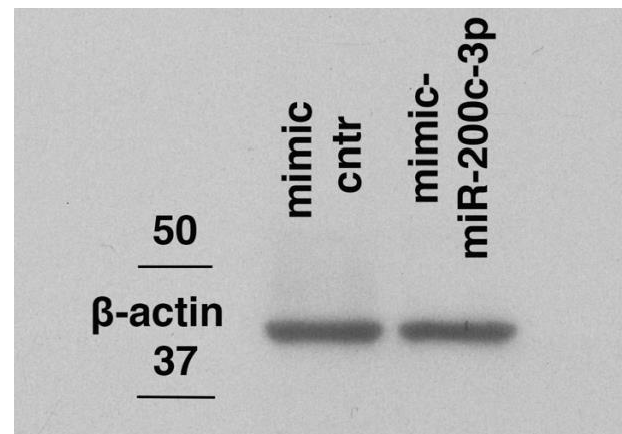

Supplement: S1 Raw images — (PDF) [file pone.0257070.s003.pdf]
