# Supplementary material for: MiR-200c-3p maintains stemness and proliferative potential in adipose-derived stem cells by counteracting senescence mechanisms
Source: PLoS One. 2021 Sep 17;16(9):e0257070. doi: 10.1371/journal.pone.0257070 (PMC8448302; doi:10.1371/journal.pone.0257070)
Supplement: S1 File — (DOCX) [file pone.0257070.s002.docx]

# **Supplementary Material and methods**

## **Flow cytometry**

Cells at passage 3 were subjected to flow cytometric analyses by using a FACSCalibur cytometer (BD Biosciences, San Jose, CA, USA), as previously described (PMID: 22526170; PMID: 29535768). Briefly, 0.5x10^3^ cells were harvested, centrifuged, and fixed for 30 min in ice-cold 2% paraformaldehyde. The single-cell suspensions were washed in flow cytometry buffer containing PBS, 2% FBS and 0.2% Tween 20, then incubated for 30 min with monoclonal antibodies to CD29, CD34, CD44, CD45, CD90, and CD166, conjugated to fluorescein isothiocyanate, phycoerythrin, or phycoerythrin-Cy5 (BD Biosciences). All monoclonal antibodies were of the IgG1 isotype. Nonspecific fluorescence was determined by incubating the cells with conjugated mAb anti-human IgG1 (DakoCytomation, Glostrup, Denmark).
